# A 7T fMRI investigation of hand and tool areas in the lateral and ventral occipitotemporal cortex

**DOI:** 10.1371/journal.pone.0308565

**Published:** 2024-11-05

**Authors:** Ineke Pillet, Begüm Cerrahoğlu, Roxane Victoria Philips, Serge Dumoulin, Hans Op de Beeck

**Affiliations:** 1 Department of Brain and Cognition, Leuven Brain Institute, KU Leuven, Leuven, Belgium; 2 2LPN (Laboratoire Lorrain de Psychologie et Neurosciences de la Dynamique des Comportements), Université de Lorraine, Nancy, France; 3 Department of Cognitive and Behavioral Sciences, University of Luxembourg, Esch-sur-Alzette, Luxembourg; 4 Spinoza Centre for Neuroimaging, Amsterdam, Netherlands; 5 Computational Cognitive Neuroscience and Neuroimaging, Netherlands Institute for Neuroscience, Amsterdam, Netherlands; 6 Experimental and Applied Psychology, Vrije University Amsterdam, Amsterdam, Netherlands; 7 Experimental Psychology, Helmholtz Institute, Utrecht University, Utrecht, Netherlands; Juntendo University, JAPAN

## Abstract

Previous studies demonstrated the existence of hand and tool areas in lateral and ventral occipitotemporal cortex (OTC), as well as an overlap between them. We reinvestigated this organization using 7T fMRI, benefiting from a higher signal-to-noise ratio than 3T. This enabled us to include a wider array of categories to achieve a more holistic perspective, and to omit certain spatial preprocessing steps. Despite these improvements, univariate analysis confirmed the existence of hand-tool overlap across OTC, which is striking given the omission of the spatial preprocessing steps that can influence overlap. There was significantly more overlap between hands and tools, compared to other overlap types in the left hemisphere of OTC. The overlap was also larger in the left lateral OTC as compared to the right lateral OTC. We found in all hand areas a differentiation between tools and other types of objects, although they still responded more to bodies than to tools. Regarding the tool areas, we observed a differentiation between hands and other categories such as faces and non-tool objects. Left hemisphere tool areas also differentiated between hands and bodies. When excluding the overlapping voxels from the hand and tool areas, they still showed a significant response to tools or hands (compared to objects or faces) respectively. Multi-voxel pattern analysis indicated that neural representations in the hand areas showed greater similarity between hands and tools than between hands and other objects. In the tool areas, the neural representations between tools and hands and between tools and other type of objects were all equally similar. To summarize, capitalizing on the benefits of 7T fMRI, we further substantiate the evidence in favor of hand-tool overlap in several regions of occipitotemporal cortex.

## Introduction

In both the lateral and ventral occipitotemporal cortex (OTC), areas selectively responding to specific visual categories have been identified, for example to faces [1, 2], to bodies [3, 4], to scenes [5, 6], to words [7–9], to hands [10], and to tools [11, 12]. More specifically, the hand area, found by Bracci and colleagues (2010) [10], was located in the lateral OTC; it could be separated from the extrastriate body area (EBA) adjacent to it; and this hand area, in contrast to EBA [3], was lateralized to (i.e., responses were stronger in) the left hemisphere. In this study, they could not locate any other hand areas within the OTC that preferred hands more than bodies. However, in later research, they did find responses to hands also in right lateral OTC and in left and right ventral OTC [13]. Selectivity to hands is also found beyond OTC, in parietal regions for example [13, 14].

In their 2010 study [10], Bracci and colleagues observed hand selectivity in the left lateral OTC, which they suggested might be proximal to responses to tools in the OTC, previously reported by for example Chao and Valyear and their colleagues [15, 16]. This selectivity to tools was mostly lateralized to the left side of the brain [e.g., 17, 18], like the responses to hands found in the lateral OTC. In addition, other studies have identified regions within the OTC that selectively respond to tools, located along both the left and right medial fusiform gyrus in the ventral OTC [13, 17, 18], which seemed to process the shape and structure of tools [19]. Lesion studies revealed that these areas process functional information about manipulable objects but are not essential for object manipulation [20–22]. Like hand selectivity, tool selectivity goes beyond the OTC and is present for example in frontoparietal regions [23, 24].

Several different research groups have suggested that a part of the hand and tool areas in the left lateral OTC overlap with each other [11, 12, 14, 25–28]. These responses to tools and hands were distinct from nearby object and motion areas, and the overlap was specific to hands and tools; it did not extend to bodies [11]. Subsequent research provided clear evidence of a partial overlap between hand and tool responses in the right lateral OTC, as well as in the left and ventral OTC [13]. This overlap within OTC, a part of the visual cortex, was unexpected, given that hands and tools do not share similar visual characteristics. The organization of OTC is determined by several dimensions on which categories vary, for example in ventral OTC, selective response to animate categories appeared more lateral than to inanimate categories [29]. Other dimensions, such as form [30] and manipulability [17], fail to explain the overlap because hands and tools differ in these aspects [25]. Thus, several studies attempted to understand why the hand and tool area in left lateral OTC would overlap. Several paths have been investigated, such as common visual features [for a discussion see e.g., 12] and visual imagery [for a discussion see e.g., 11]; or action-related characteristics like hand-centrality and body extension [for a discussion see e.g., 14]. For this, studies used several paradigms, such as fMRI adaptation and TMS, and tasks such as real tool grasping [25, 27, 28], in the healthy population but also in the congenital blind [e.g., 12] and people without hands [e.g., 26]. (Functional) connectivity have also explored the reasons for this overlap [e.g., 11, 31].

In our recent 7T fMRI study that focused upon the functional neuroanatomy of word selectivity [32], we could locate the hand areas found in left and right lateral and ventral OTC. This finding suggests that our dataset can reassess hand and tool selectivity and their overlap, addressing limitations of previous studies. The aforementioned studies on the overlap between hand and tool responses in lateral and ventral OTC were limited by the use of 3T or 4T fMRI and thus a standard spatial resolution [e.g., 10, 11, 13]. A higher spatial resolution may influence our understanding of the overlap between areas, shedding light on the intricacies of their organization and selectivity [11, 28, 33]. As an example, prior research questioned if separate selectivity to faces and bodies existed within the fusiform gyrus or if they overlapped completely [33]. When investigated under a higher spatial resolution, it became evident that while these areas exhibit some degree of overlap, distinct face and body regions could be discerned [33]. Similarly, Caffarra and colleagues (2021) argued that regions of activity, such as the visual word form area, might be subdivided when examined with high anatomical precision [34]. It is important to note that spatial preprocessing steps can also influence overlap between areas. Prior studies on the overlap between hand and tool areas have often preprocessed the data by spatial normalization and smoothing [e.g., 11, 13]. Omitting these preprocessing steps allows the data to remain faithful to the gyral and sulcal patterns of individual brains, facilitating more accurate localization of activity [35]. Spatial smoothing for instance could lead to averaging activity together that on the surface are in truth some distance away from each other [35]. Therefore, we sought out to reinvestigate the hand and tool responses and their possible overlap in occipitotemporal cortex, aiming for a more holistic perspective by including a wide array of categories, omitting these spatial preprocessing steps and using a modestly increased spatial resolution, made possible by the higher signal-to-noise ratio that 7T fMRI provides compared to 3T [36, 37]. In our study, we defined left and right lateral and ventral hand and tool areas and investigated the specificity of their overlap and possible differences in overlap between regions of the OTC, using univariate analysis. We then examined the response profiles of these areas and of the non-overlapping hand and tool areas to determine if these were exclusively selective. In addition, we examined their representational space using multi-voxel pattern analysis (MVPA). We also identified face, body, and object areas as a comparison/control in our univariate analyses and included these categories (and several others) in the representational space as a reference.

## Methods

Some parts of this methods section contain information previously described in another study that also utilized this dataset [32].

### Participants

Nineteen participants took part in this research (mean age: 30.1±6.8 (23–45), sex: 11 males, 8 females). Sixteen of them were right-handed and the other three were left-handed (subject 2, 7 and 17). The participants all had normal or corrected to normal visual acuity. They all provided written informed consent. The study was approved by the ethics committee of Vrije Universiteit Amsterdam and followed the guidelines of the Declaration of Helsinki (approval number: VCWE-2020-004). Recruitment started the 1^st^ of March 2020 and ended the 5^th^ of September 2020.

### Stimuli

Stimuli (500x500 pixels, 4.7 degrees visual angle) appeared on a 32ʹʹ LCD screen (69.8 × 39.3 cm, 120 Hz) that was designed for an MRI environment (BOLDscreen, Cambridge Research Systems, UK). The resolution of the screen was 1920 × 1080 pixels. It was positioned at the end of the bore and participants viewed it through a mirror (distance from screen: 220 cm) that was mounted on the head coil. Stimuli were presented using MATLAB (MathWorks, Inc.) and the Psychophysics Toolbox Version 3 [38–40]. Stimuli were presented at a semi-random location around the middle of the screen, with the center of stimulus maximum 33 pixels/0.32 degrees visual angle away. This method aimed to prevent low-level visual confounds (see also the SHINE toolbox manipulation described below) and to increase the difficulty of the one-back task. fixation dot was always present in the middle of the screen, appearing over the stimuli whenever they were presented.

**Fig 1:**
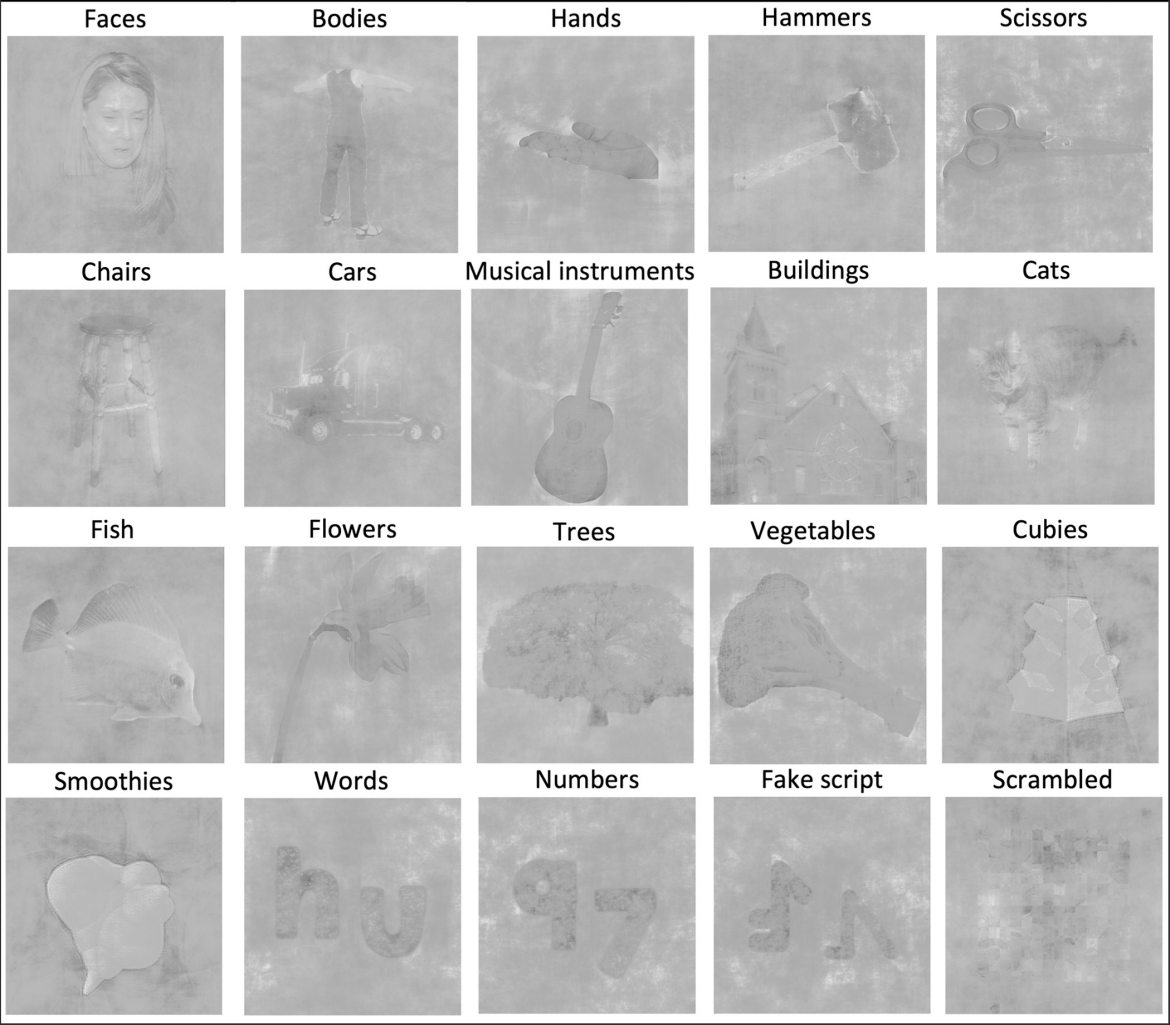
The stimuli of the experiment. An example stimulus of every condition. The condition that it belongs to is written on top of the image.

The experiment was designed so that it could be interesting for different research aims. Twenty conditions were selected, including 19 categories and 1 scrambled control condition, to ensure a diverse and comprehensive stimulus set based on various criteria. The conditions consisted of natural and artificial objects, familiar and new shapes (cubies and smoothies) [41], animate and inanimate shapes, objects that differ in real-world size, objects differing in how they are used and whether they are a tool or not, … These dimensions allow for flexible grouping of conditions based on the exact goals of the research. For every condition, we included different viewpoints/angles on the shapes. There was also ample variability in the identity of the stimuli in each condition. What follows is a list of all the conditions (an example image of each one is depicted in Fig 1): faces, bodies, hands, hammers, scissors, chairs, cars, musical instruments, buildings, cats, fish, flowers, trees, vegetables, cubies, smoothies, words, fake script, numbers, scrambled.

When selecting images for the conditions, we tried to minimize low-level and mid-level feature differences between the categories. For eight (bodies, buildings, cars, cats, chairs, faces, fish, hammers) of the 20 conditions, Michael Cohen [42] provided the images. They ensured high within-category variability by for example, using images of objects at different angles, at different positions, and so on [42]. We followed similar procedures for selecting images for the remaining conditions. The following conditions (partially) consist of stimuli from the Bank of Standardized Stimuli (BOSS): musical instruments, flowers, vegetables, scissors [43]. To have more stimuli for these conditions and to have stimuli for the other categories (hammers, hands, trees), we used freely available images on the internet.

We used the randblock(rgb) function on all the stimuli (available online for MATLAB (Mathworks, Inc.)) to create a control condition (scrambled images). We then selected 30 scrambled images and included at least one stimulus from each of the other 19 conditions. These 30 images contained more variability in the middle than at the borders. This resembles the general organization of the stimuli of the other conditions.

As new shapes, we used the cubies and smoothies categories as described by Op de Beeck and colleagues (2006) [41]. The shape of both categories varied on four different dimensions. On each of the dimensions, the shape was rated from 0 to 5. We selected 16 shapes, both for cubies and for smoothies, that had an extreme position in this 4D-space (e.g., 0505, 0005) and four others that had a center position within this space (e.g. 2222, 2323). Ten of those 20 stimuli were duplicated before any further processing happened, resulting in a total of 30 stimuli. All 30 were then flipped at a semi-random angle: the duplicates were angled differently than their original version so that we would have 30 unique stimuli. We ensured that the positions of cubies and smoothies on the grey background varied and were not always centered.

The words, the fake script and the numbers conditions consisted of two, three or four letters/characters. These letter/symbol strings were angled ascending or descending to vary the aspect ratio among stimuli within the conditions. The letters/symbols were in a bold font that was filled with a random dotted pattern. Half of the images of each condition were placed onto a white background and the others on a black background. These characteristics enhanced comparability of these conditions with others in terms of retinotopic envelope. The words had no semantic meaning; however, they all contained at least 1 vowel (except one of the 30 stimuli) and were all pronounceable. For simplicity, we refer to this category as ’words’ rather than ’letter strings’ or ’pseudo-words’. To create the fake script, we used a combination of two different fonts so that the letters would take on variable shapes (e.g., short, long, blocked, curvy).

On top of controlling variability within conditions in viewpoints and identity of the stimuli, some of the conditions required extra controls to match their low and mid-level features to that of the other categories. Since hammers and scissors often have an elongated shape, we translated and rotated these stimuli and made sure to include both open and closed scissors, to avoid retinotopic confounds. For the trees, since they have a very specific shape, we used different kinds of trees and also resized them (smaller/larger). We also made sure that they were not always located on the middle section of the background but also on the upper/lower left/right. In the stimuli of the buildings, we replaced the images that included trees, with other stimuli of buildings without trees, because trees is a different condition. The stimuli of the bodies were also edited so that no hands would be visible, since this is also supposed to be a different condition.

For every condition, there were 30 stimuli available. All these images were then matched for average luminance, contrast and spectral energy using the SHINE toolbox [44]. This procedure was inspired by the study of [42]. We ensured that after this manipulation, the identity of the stimuli was still at least moderately clear and visible. Additionally, we conducted further controls by generating mean greyvalue, standard deviation greyvalue, and edge images per condition, and compared them across conditions. By doing this, we ensured that the conditions did not differ on these measures.

All images, and in addition all data (including raw data of all participants in the surface space) relevant to the analyses presented in this study, are available in the following GIN repository: https://doi.org/10.12751/g-node.96eqnl [45].

### Experimental design

Before entering the scanner, the participants received instructions for the task they would be doing once inside. They had to keep their focus on the fixation dot in the middle of the screen throughout the whole task. They had to perform two tasks at once. The first was a one-back task where they had to press a button with their left or right hand (this changed every subject) when an image on the screen was an identical copy of the previous image. Participants were instructed not to press the button if a stimulus was presented again but rotated or angled differently, as it was not an identical copy of the previous stimulus. In the second task, participants pressed a button in the other hand than the hand used for the first task, as soon as possible when they perceived a change in category. This second task was used so category information would also be processed, and participants could not only focus on more low-level features. To facilitate performance of the second task, participants were introduced to all categories by presenting an example stimulus from each category. Participants achieved a high average score in the category task, with a hit rate of 85% and a false alarm rate of 1%. In contrast, scores on the one-back task were lower, with a hit rate of 65% and a false alarm rate of 10%. This was expected given the fast pace of the stimuli presentation in a block (one every 0.67 seconds).

In total, the participants went through seven runs of the experiment inside the scanner (in one subject only six, some subjects completed eight). Each run consisted of 40 blocks lasting 10.05 seconds each. Each included 15 trials (i.e., stimuli). On top of those, 3 extra blocks were added (at the start, middle and end of the run) that contained only a fixation dot for 15 seconds. One trial took 0.67 seconds: during 40% of the trial (0.27 seconds) only the fixation dot was present, for the other 60% of the trial (0.40 seconds) there was a stimulus present. The stimuli in a block were always presented in a random order, and two repeats of stimuli (for the first task) happened at random times within a block. One run lasted 447 seconds. The first 20 experimental blocks (for the 20 conditions) were presented in a random order. Following the middle fixation block, the remaining 20 blocks appeared in the reverse order compared to the first 20 blocks. This resulted in 140.7 seconds of data collected for each category in each subject across 7 runs. We used two types of runs: the 15 images for each block/condition came from the first (type 1) or the second half (type 2) of the full collection of 30 images available for that condition.

### (f)MRI acquisition

Data was collected using a 7T Philips Achieva MRI scanner (Philips Healthcare, Best, The Netherlands) and an 8 channel transmit coil and a 32 channel receive coil (Nova Medical Inc, Wilmington, United States) at the Spinoza Centre for Neuroimaging in Amsterdam (the Netherlands). Universal pulses were used during scanning [for explanation see 46]. During scanning imaging, we used a respiration belt and a pulse oximeter on a finger of the left hand to collect respiratory and cardiac data.

To collect the functional images, we used a 3D-EPI sequence with the following parameters: volume repetition time (TR) = 1.37 s, echo time (TE) = 16.9 ms, flip angle = 13°, voxel size = 1.79x1.79x1.8 mm, field-of-view (FOV) = 200x200x176 mm and matrix size = 112 x 112 x 98. We also acquired images using an identical sequence but for reversed phase-encoding blips i.e. phase-encoding in the opposite direction. Those images were later used to correct for distortions. To collect anatomical images, we used a MPRAGE sequence with parameters: TR = 10 ms, TE = 3.3 ms, flip angle = 8°, spatial resolution = 0.8x0.8x0.8 mm, matrix size = 288x288x205.

### Preprocessing

#### (f)MRI data

The data was initially converted to NIfTI files using DCM2NIIx [47] and reoriented to RAS+ orientation using nibabel in Python. Subsequently, the data was formatted according to the Brain Imaging Data Structure (BIDS) [48]. Preprocessing was performed through fMRIPrep 20.2.0 [49, 50] (RRID:SCR_016216), which is based on Nipype 1.5.1 [51, 52] (RRID:SCR_002502). In summary, the functional images were corrected for susceptibility distortion, realigned and coregistered to the anatomical image. In addition, the brain surface was reconstructed based on the anatomical image with Freesurfer, as implemented in fMRIprep. Spatially normalized data was not utilized to retain individual participant brain space. This way, we could keep as much spatial and anatomical detail as possible. In its report, fMRIprep provided details on the preprocessing steps taken and this description can be found below. To assess fMRI data quality, tSNR images (average of the time series divided by the standard deviation) for each participant were examined.

During data collection, there was a temporary issue with the coil resulting in darker imagery in the right hemisphere compared to the left in some subjects (subjects 4 to 10). In the anatomical data, such a shadow could affect the surface reconstruction. To avoid this problem, we went through several steps. For each anatomical scan, we looked at the distribution of intensity values using ITK-SNAP [53] and then clipped them accordingly; then we rescaled those values using ANTs [54]; and then we performed denoising and bias correction using SPM12 and cat12. The surface reconstruction results from a fMRIprep anat only process were then carefully checked and improved where needed. All participants’ brain surfaces were successfully reconstructed. After all these steps we ran fMRIprep in full to also perform the functional preprocessing steps while making use of these anatomical results.

The shadow did not cause trouble in the functional preprocessing. We carefully checked the preprocessing results using the fMRIPrep reports and through checking if several contrasts (e.g. faces versus all other conditions) were of similar strength and size with (1) a general linear model based on the functional space after realignment, versus (2) a general linear model after realignment, susceptibility distortion correction and coregistration to T1w space, versus (3) a general linear model after all these steps plus normalization to MNI space. This check was done to ensure that all preprocessing was successful.

A detailed description of the preprocessing steps, provided by fMRIPrep can be found in the supplementary material (S1 Text. Supplementary Methods).

#### Physiological data

Using scanphyslog2bids created by Lukas Snoek (available through GitHub (https://github.com/lukassnoek/scanphyslog2bids), we formatted the physiological data according to BIDS [48]. We corrected for physiological noise using RETROICOR [55, 56] which included using Fourier expansions of different order for the estimated phases of cardiac pulsation (3^rd^ order), respiration (4^th^ order) and cardio-respiratory interactions (1^st^ order) [57]. The corresponding confound regressors were set up using the MATLAB PhysIO toolbox [58] which is open-source code available as part of the TAPAS software collection (https://www.translationalneuromodeling.org/tapas). The physiological data collection did not succeed for all participants: participants 2, 3, 4, 5 and the first functional run of participant 18 were without physiological data.

### Data analyses

#### Univariate analysis

Using the Statistical Parametric Mapping toolbox (SPM12) in MATLAB, we constructed a general linear model (GLM) for each participant, modeling every voxel per run. Each condition was modelled by a regressor with certain onsets and a certain duration. This resulted in 21 regressors, comprising 20 categories and one fixation condition. Additionally, we included several confound regressors from fMRIprep and the PhysIO toolbox. Specifically, we used the realignment parameters and their temporal derivatives (total: 12) to minimize motion effects on the data. For each participant and run, fMRIPrep also computed a motion outliers regressors (a binary regressor for each volume in the run labeled as outlier) and these were also added to the GLM. The number of outliers varied across runs and participants. When respiratory and cardiac data were available, the resulting confound regressors (total: 18) were also added to the model. We applied a high-pass filter with a cutoff of 610 seconds, based on the run’s design. Each condition was contrasted versus the average of all other conditions excluding the fixation condition (which we will refer to as ‘all’ for simplicity) to detect the voxels that respond more to that condition than to all the others. For the several goals of this study, we were interested in particular in the following categories’ beta values: hands, hammers, scissors, bodies, faces, chairs, cars, musical instruments, building; and in the following contrasts: hands vs all, tools (hammers and scissors) vs all, bodies vs all, faces vs all, chairs vs all, cars vs all, musical instruments vs all and buildings vs all.

Our first goal was to visualize specific contrasts’ results on the brain surfaces of our participants. To do this, we selected the results from hands vs all remaining categories except fixation, tools (hammers and scissors) vs all, bodies vs all, and faces vs all. For clarity of the visualization, we only selected one typical object category contrast: chairs vs all. The end result will be depicted by a figure where the result of these contrasts is shown on the reconstructed brain surface of example subjects. Contrasts were family-wise error corrected with a significance level set at *p* < 0.05.

As previously stated, all analyses were conducted within the individual brain space without spatial normalization. This approach is in line with the recommendation of Weiner and Grill-Spector (2013) [35], summarized in their Table 2. It preserves the gyral and sulcal patterns of each individual brain, facilitating more accurate localization of activity. Furthermore, they advise against spatial smoothing due to its potential to distort activity localization and average distant regions on the surface. We adhere to the procedure outlined by Brodoehl et al. (2020) [59], where the GLM is established for each participant using data in its original, unnormalized, and unsmoothed volume-space. These results can be projected onto the brain surface of each participant separately.

**Fig 2:**
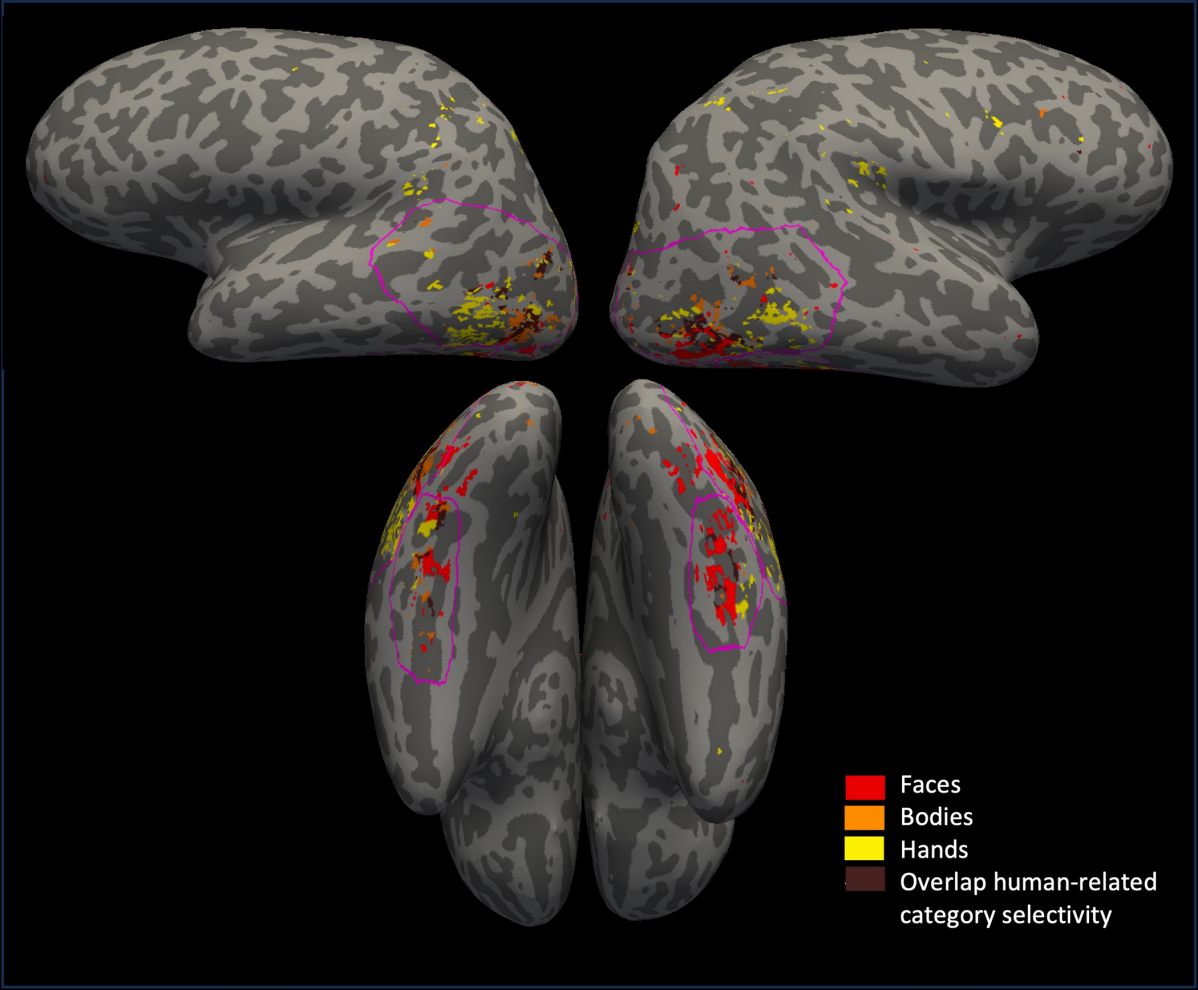
The anatomical regions of interest. The anatomical regions of interest (ROI) for both the left lateral, left ventral, right lateral, and right ventral hemisphere, shown using a pink outline on a brain surface of one of the participants. Selectivity to faces shown in red on the surface, bodies in orange, hands in yellow, and any kind of selectivity overlap between these three categories in brown.

For each participant, we also created four anatomical ROIs. We visualized significant activity to faces, bodies and hands on the brain surface. We then manually drew four ROIs around this activity within the occipital and temporal cortex: a lateral left ROI, a lateral right ROI, a ventral left ROI and a ventral right ROI (see Fig 2).

Then, these four anatomical ROIs were intersected with the contrasts’ results from the GLM to create anatomical-functional ROIs (20 in total) using five different contrasts: hands vs all, tools vs all, bodies vs all, faces vs all, and chairs vs all (*p* < .05 with FWE correction). This process allowed us to define for example, a left lateral hand area, a right ventral tool area, … Not all anatomical-functional ROIs could be created at this significance level for every participant. At this step, we also defined ‘exclusive’ hand and tool areas (i.e. areas excluding voxels that overlapped between the hand and tool areas): areas selective to hands vs all but not significantly selective to tools vs all, and areas selective to tools vs all but significantly selective to hands vs all.

Three of the participants (2, 7 and 17) were left-handed. However, compared to the right-handed participants, their organization of selectivity was similar, and overall, they showed lateralization of hand and tool selectivity to the same extent. A similar tool organization in left-handed and right-handed has been demonstrated before [60]. Only participant 17 showed some evidence for a possible reversal of lateralization (i.e., to the right instead of to the left hemisphere, see S5 and S6 Figs). Due to the overall similarity between the left- and right-handed participants, they were all included in the analyses.

Next, the number of voxels in each anatomical-functional ROI was determined (subjects without a particular ROI were excluded from analysis). Additionally, voxel overlap between hands and tools, faces and tools, bodies and tools, and hands and objects was calculated. For each of these overlap types, we divided the number of overlapping voxels by the total of voxels in those ROIs and transformed this into a percentage. The average percentages per overlap type for lateral/ventral left/right OTC were then visualized in a bar graph.

Subsequently, several statistical tests were conducted. First, in left lateral, right lateral, left ventral and right ventral, we compared the percentage of overlapping voxels (again divided by the total of voxels for both categories) between hands-tools and faces-tools, between hands-tools and bodies-tools, and between hands-tools and hands-objects (significance was set at *p* level < .01 based on Bonferroni correction). Second, we also compared the percentage overlap of hands-tools (divided by the total number of voxels for those two categories) between lateral and ventral in both the left and the right hemisphere and between the left and right in the lateral and ventral OTC (significance level set at *p* < .01 based on Bonferroni correction). The results of these tests are also depicted in the bar graph.

Then, to examine the response profiles of all the created anatomical-functional ROIs, we selected the beta values from the GLM (explained at beginning of this section) for hands, hammers, scissors, bodies, faces, chairs, cars, musical instruments and buildings, for all the voxels within the ROI and per subject, and then averaged these beta values (per category) across all runs and all voxels within the ROI. As a result, we ended up with one beta value per category, per ROI and per subject. Then, we averaged across subjects to create a bar graph depicting the averaged beta value per category per ROI and calculated the standard error for each category/bar. These bar graphs do not allow us to draw conclusions about the beta value of the category used to define thr anatomical-functional ROI. For example, the left lateral hand area was created by an intersection between the left lateral anatomical region and the results from the contrast hands vs all, selecting only the voxels that were present in the anatomical ROI and were significant (FWE correction *p* < .05) in this the contrast. As a result, all the voxels that make up the anatomical-functional ROI ‘left lateral hand area’ are biased towards a high beta value for hands and thus no conclusions should be drawn within this area about the category ‘hands’.

Within each anatomical-functional ROI, several statistical tests were conducted to address the aims of this study. For the four hand ROIs, paired t-tests were conducted to test if the response to the tools category (hammers and scissors) was higher than to (1) non-tool objects (chairs, cars, and musical instruments, excluding buildings), (2) bodies, and (3) faces. (significance level was set at *p* < .02 based on Bonferroni correction for 3 tests, per ROI). In the tool ROIs, we tested if the response to hands was significantly higher than to (1) objects (chairs, cars and musical instruments), (2) bodies, and (3) faces (significance level was set at *p* < .02 based on Bonferroni correction for 3 tests, per ROI). In the other ROIs (faces, bodies and chairs), we compared the response to tools with objects (chairs, cars and musical instruments, except for chairs in the case of the chairs ROIs). The significance level was set at *p* < .05 for this test, since only this one test was performed in these ROIs.

In addition, we investigated the exclusive hand and tool areas (i.e. without including the overlapping voxels between these hand and tool areas), to determine if these exclusive areas also showed a significant response to tools or hands (compared to other categories). After excluding the overlapping voxels of the four hand and tool areas in every participant, the beta values of all remaining voxels to tools, non-tool objects, faces and bodies in the hand areas, and to hands, faces, bodies and non-tool objects in the tool areas, was selected from all the runs in the full GLM in each ROI. These beta values were then averaged first across runs and then across all voxels in the ROI. In the case of the tool and non-tool conditions, we then averaged across hammers and scissors or cars, chairs and musical instruments respectively. Using a paired t-test, in the hand areas, the response to tools was compared to non-tool objects, faces and bodies, and in the tool areas, the response to hands was compared to faces, bodies and non-tool objects. If a participant did not have a certain ROI, they were excluded from this test. Significance level was set at *p* < .02 Bonferroni corrected per ROI.

#### Multi-voxel pattern analysis

To conduct a multi-voxel pattern analysis within the left/right lateral/ventral hand and tool areas, we split the dataset of every participant into two. We used the anatomical ROIs drawn on the surface of every subject for the univariate analyses (see Fig 2). Then, these four ROIs per subject were intersected with the results from a GLM containing all odd runs of the dataset of that subject, for the contrast hands vs all and tools vs all (uncorrected *p* < .0005). This process is similar to the univariate analysis, except that in the univariate analysis, we intersected with a GLM based on all runs. This resulted in 8 anatomical-functional ROIs per participant. Some ROIs could not be created due to the absence of voxels that reached significance.

Then, using the GLM containing the even runs for every participant, we constructed a multi-voxel pattern in response to each of the conditions for each run, by using the beta coefficient estimates of every voxel that was included in the ROI. We repeated this for each of the 8 ROIs. We used the CoSMoMVPA toolbox [61] for MATLAB (Mathworks, Inc.). To decode the category for every possible pair of categories (excluding the fixation condition), for example hands-faces, tools-faces, …, we used the cross-validated Mahalanobis distance [62], also termed the linear discriminant contrast (LDC). The code for that was written by J. Brendan Ritchie. We then saved the results in a dissimilarity matrix or in this case more specifically, a distance matrix, where each point in the matrix showed the distance between the two categories of that row and column. This distance represented a dissimilarity between the multi-voxel pattern between those two categories in that ROI. The higher the distance, the more dissimilar the patterns were. We created distance estimates for every category pair in each ROI by using a cross-validation process where the dataset is split up into training and testing partitions, according to the standard leave-one-run-out partitioner. By this process, each run was used once as a test while the other runs were used as training. As a result, there were as many distance estimates as there were runs for that participant. We then averaged across these estimates and placed the average inside the dissimilarity matrix of that participant and ROI. Every participant matrix for each ROI was normalized by dividing all the values inside by the maximum value of that matrix. Then, per ROI, we averaged across all participants’ matrices. We computed the reliability of these matrixes by correlating every participant’s matrix with the average matrix (calculated excluding that subject). Finally, we performed several paired t-tests (using a Bonferroni corrected *p*-value) between different distances.

Multidimensional scaling (MDS) was used to visualize the main dimensions underlying the patterns in the representational dissimilarity matrices in a two-dimensional space where the distance between the points in this space was a measure for how dissimilar these points were: the higher the distance, the more dissimilar. We used the built-in MATLAB (Mathworks, Inc.) function mdscale with the default parameters, while minimizing the default goodness-of-fit criterion: stress and using a 100 replicates of the scaling. We applied MDS on the matrices, averaged across subjects, keeping left and right OTC separate. In addition, we also applied MDS on the normalized dissimilarity matrices of each individual participant. The MDS results of every subject, per ROI, were transformed to the average MDS results using a Procrustes transformation (using the built-in MATLAB (Mathworks, Inc.) function procrustes). We then visualized the average MDS results in a 2D space per ROI. In this space per ROI, for each category, 19 lines were drawn using the Procrustes transformed individual subject MDS position of that category. The line started in the dot of a category and ended at the coordinates (Procrustes transformed MDS results) of that category for the individual participant.

## Results

### The overlap between hand and tool areas

**Fig 3:**
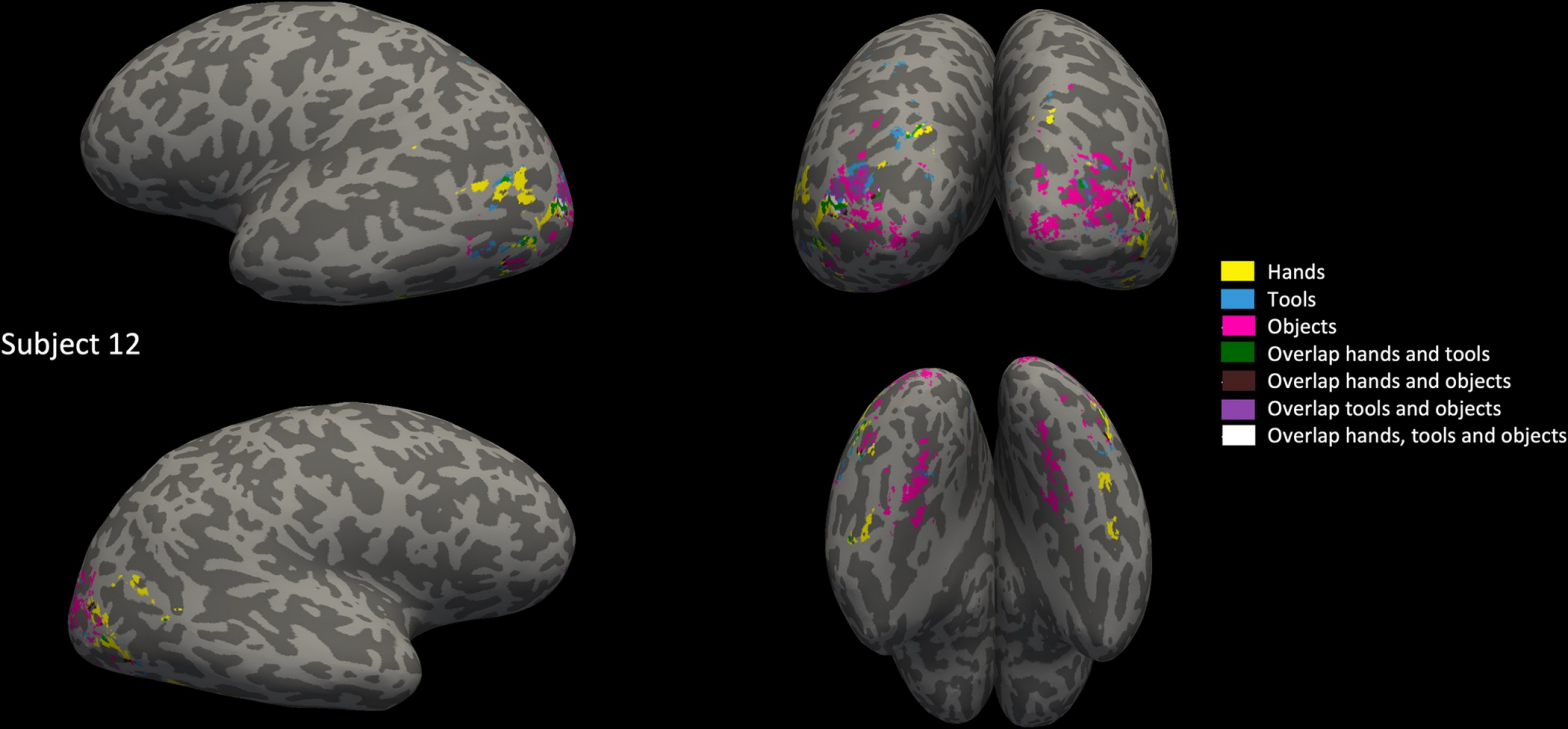
Hand, tool and object areas and their overlap. Hand, tool and object areas and their overlap, determined by a contrast of one versus all other categories except fixation, p < 0.05, FWE corrected (hands in yellow, tools in blue, objects (chairs) in pink, overlap between hands and tools in green, between hands and objects in brown, between tools and objects in purple and between hands, tools and objects in white), shown upon annotated left lateral (top left), posterior (top right), right lateral (bottom left) and ventral (bottom right) brain surface of one example participant (color legend also on the right of the figure).

**Fig 4:**
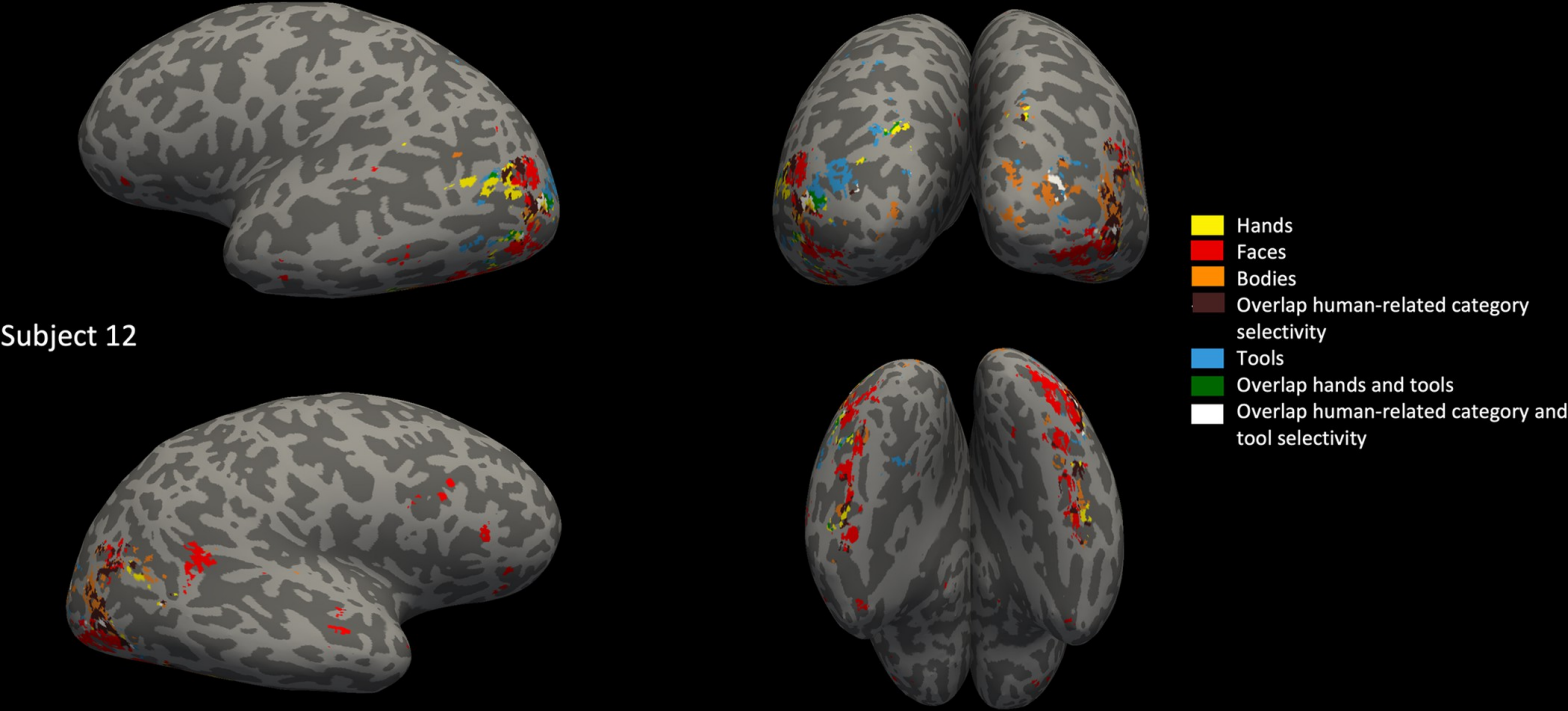
Hand, face, body and tool areas and their overlap. Hand, face, body and tool areas and their overlap, determined by a contrast of one versus all other categories except fixation, p < 0.05, FWE corrected (hands in yellow, faces in red, bodies in orange, overlap between some or all of these three animate categories in brown, tools in blue, overlap between hands and tools in green, and between animate categories and tools in white), shown upon annotated left lateral (top left), posterior (top right), right lateral (bottom left) and ventral (bottom right) brain surface of one example participant (color legend also on the right of the figure).

In a previous study, the overlap in left lateral OTC between hand and tool areas was quantified and compared to overlap with another category (bodies) to show the specificity of this overlap [11]. In a later study, overlap was also observed between the hand and tool areas in other parts of OTC, but was not tested against other categories [13]. In this analysis, we aimed to provide a quantification and test of hands-tools overlap specificity beyond left lateral OTC, overcoming several of the drawbacks of previous studies that could have influenced their results. We modelled activity to the experimental conditions using a GLM for each subject and then determined several types of category selectivity (faces vs all conditions (except the fixation condition), bodies vs all, hands vs all, tools vs all, objects: chairs vs all) on the individual’s brain surface. We found selectivity to faces, bodies, hands, tools and objects in every subject (see Figs 3 and 4 for an example subject, see S1–S8 Figs for more example subjects). In each anatomical ROI that we drew per subject (left/right lateral/ventral OTC), we selected only the voxels significantly (*p* < .05, FWE correction) active to hands/tools/bodies/faces/objects: chairs respectively.

**Fig 5:**
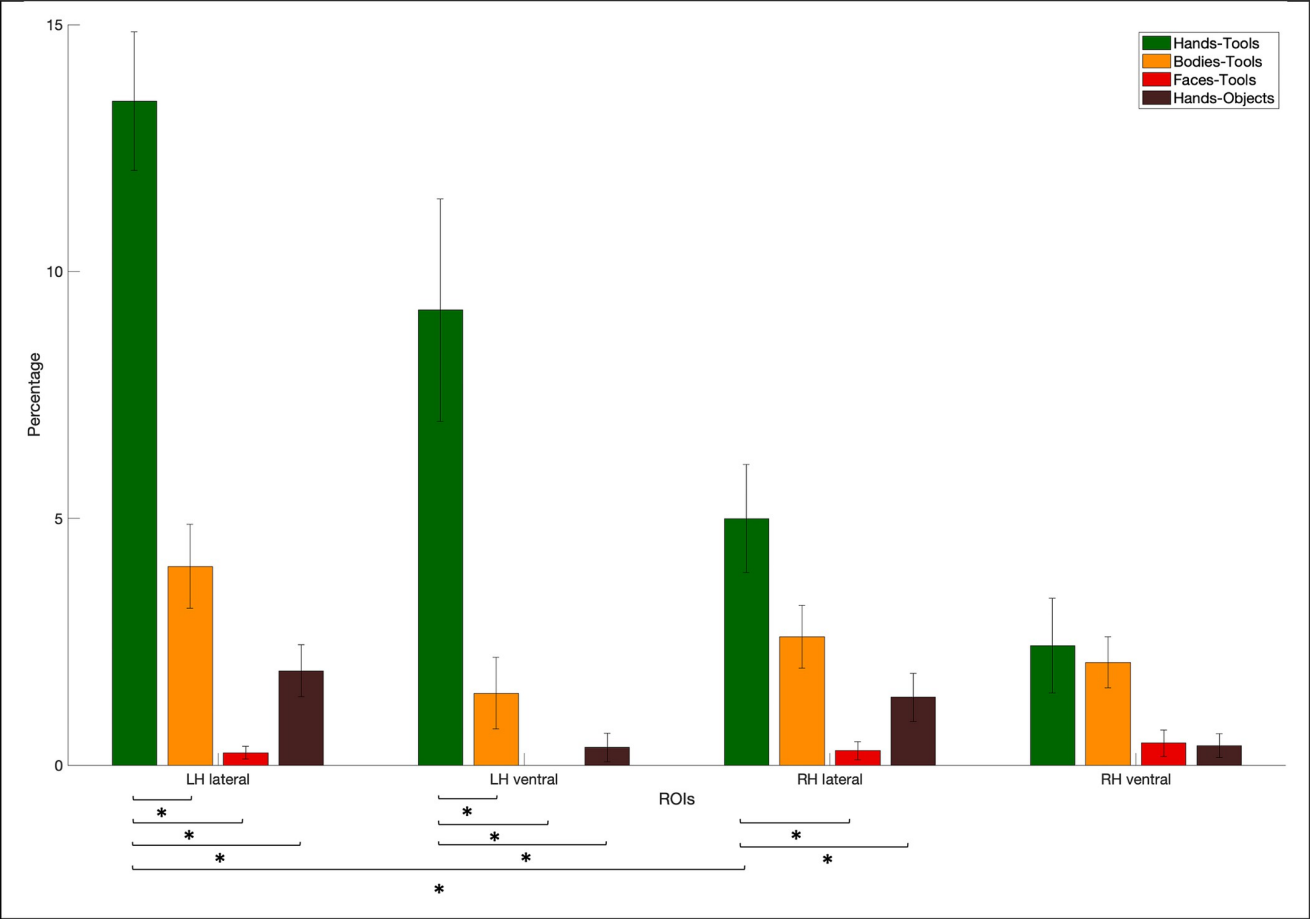
The average percentage of overlap for different overlap types. The average percentage of overlap between hands and tools (green), between bodies and tools (orange), between faces and tools (red) and between hands and objects (chairs, brown). Statistically significant tests between types of overlap and between different regions (left/right lateral/ventral OTC) were indicated by a *.

We specifically analyzed the size of the overlap between selective areas, by calculating the size of different types of category overlap, normalized for total size by dividing by the total size of the two category areas in that type of overlap (results are depicted in Fig 5). We tested if the hands–tools overlap was significantly larger (i.e., *p* < .01 based on Bonferroni correction) than bodies–tools, than faces–tools, and than hands–objects (chairs). In left lateral and in left ventral OTC, the hands–tools overlap was significantly larger than bodies–tools (left lateral: *t*(17) = 5.62; *p* = 3.02*10^−5^; left ventral: *t*(16) = 3.16; *p* = .006), than faces–tools (left lateral: *t*(17) = 8.95; *p* = 7.66*10^−8^; left ventral: *t*(16) = 3.87; *p* = .001), and than hands–objects (chairs) (left lateral: *t*(17) = 8; *p* = 3.65*10^−7^; left ventral: *t*(9) = 3.27; *p* = .01). Note that the degrees of freedom vary because we only included subjects that showed selective voxels for both tested categories. Bracci and colleagues (2012) [11] found in their study that in the left lateral OTC, the overlap between hands and tools was larger than that between bodies and tools, consistent with our findings. As far as we know, such an analysis of overlap within other regions of the OTC had not been conducted prior to this study. In the right lateral OTC, the overlap hands–tools was not significantly larger than bodies–tools (*t*(16) = 2.47; *p* = 0.02), but it was larger than faces–tools (*t*(15) = 3.63; *p* = .002) and hands–objects (*t*(15) = 3.17; *p* = .006). In the right ventral OTC there were no differences, not with bodies–tools (*t*(6) = 0.03; *p* = .98), with faces–tools (*t*(6) = 1.13; *p* = .30) or with hands–objects (*t*(2) = 1; *p* = .43). Note however that these tests were not very meaningful in right ventral OTC because we did not have sufficient selectivity to each of the individual categories.

In addition, we tested if, separately in each hemisphere, the lateral overlap was larger than the ventral. Results revealed no significant (i.e., *p* < .01 based on Bonferroni correction) differences (*t*(16) = 1.6; *p* = .13 in the left hemisphere and *t*(6) = 0.8; *p* = .46 in the right hemisphere). We also tested if there was more overlap in the left than in the right hemisphere, separately for the lateral and ventral OTC. Results showed that in the lateral OTC, there was more hands–tools overlap in the left as compared to the right hemisphere (*t*(15) = 4.38, *p* = .0005), but this was not the case in the ventral OTC (*t*(5) = 1.8, *p* = .13).

### The response profile of hand and tool areas

In each of these anatomical-functional ROIs that were used in the overlap analysis, we also investigated the response profile of these areas to other relevant categories than hands/tools, overcoming several drawbacks of previous studies that might have influenced their results and adding new aspects (e.g., including the category faces in tests). With this analysis, we aimed to provide a more holistic understanding of the functional roles that these areas and their overlap might play in visual recognition and the action domain (see e.g., [12–14]). It’s important to note that the results from the category used to create the ROI, should not be interpreted.

**Fig 6:**
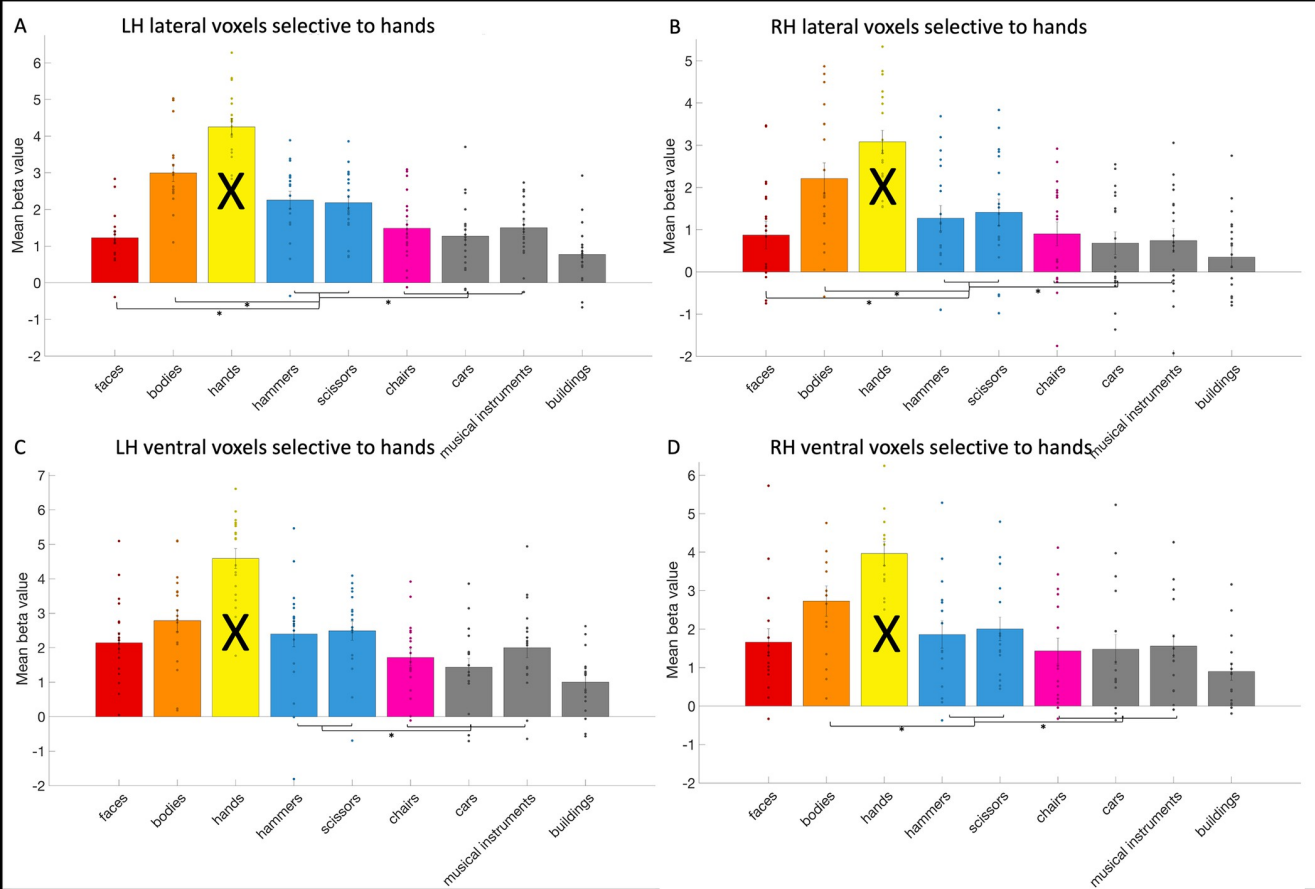
The response profile of the hand areas. The response profile on average and per subject (visualized by a dot per category bar) to faces, bodies, hands, hammers, scissors, chairs, cars, musical instruments, and buildings in the (A) left lateral (B) right lateral (C) left ventral and (D) right ventral hand areas. The results regarding to hands could not be interpreted (indicated by a black cross across the bar) as this was already used to define the hand areas. Significant statistical tests between categories were indicated using a *.

We first investigate tool selectivity in the hand areas. We could define the left lateral (mean size: 72.42 voxels, standard deviation: 38.67), left ventral (mean size: 9.16 voxels, standard deviation: 6.13) and right lateral hand area (mean size: 41.26 voxels, standard deviation: 34.33) in all 19 subjects. The right ventral area (mean size: 5.26 voxels, standard deviation: 8.02) could be defined in 15 subjects. In the left lateral hand area (see Fig 6), we observed that the activity in response to the two tools categories (hammers and scissors) was significantly (i.e., *p* < .02 based on Bonferroni correction) higher than to faces (*t*(18) = 7.4; *p* = 7.35*10^−7^) and to other objects (chairs, cars and musical instruments, *t*(18) = 3.93; *p* = .001). However, the response to bodies was higher than to the tools (*t*(18) = -3.91; *p* = .001). In the right lateral hand area, activity in response to the tools categories was higher than to faces (*t*(18) = 2.71; *p* = .01), and to other objects (*t*(18) = 6.54; *p* = 3.82*10^−6^), but lower compared to bodies (*t*(18) = -6.03; *p* = 1.06*10^−5^). In the left ventral hand area, the response to tools did not differ significantly from faces (*t*(18) = 1.09; *p* = .29) or from bodies (*t*(18) = -1.03; *p* = .2), but it was higher than to objects (*t*(18) = 3.54; *p* = .002). In the right ventral hand area, the response to tools did not differ from faces (*t*(14) = 1.16; *p* = .27), but it was lower than bodies (*t*(14) = -4.33; *p* = .0007) and higher than objects (*t*(14) = 4.02; *p* = .001).

Thus, all hand areas exhibited differentiation between tools and other objects (that included chairs, cars and musical instruments). These findings align with those reported in the left lateral hand area from Bracci and colleagues (2010) [10]. In their 2012 study two [11], they did not explicitly report if the left and right lateral hand area that they identified, differentiated tools from other objects. However, from the bar graph of their results, it would seem so in the left lateral hand area, but not in the right lateral hand area. The latter is in contrast with our results. In their study in 2016, Bracci and colleagues [13] found similar results as in their previous studies: a higher response to tools than to other objects in the left lateral hand area, but not in any of the other three hand areas. This is in contrast with our results, where all hand areas (not only the left lateral) made a differentiation between tools and other objects.

In the left and right lateral and right ventral hand areas, the response to bodies was higher than to tools. Bracci and colleagues (2010) [10] did not observe a higher response to bodies than to tools in the left lateral hand area. In Bracci and colleagues (2012) [11], study one, they used animals and not human bodies to contrast with, so a comparison is not possible. In the second study [11], a test between tools and bodies/body parts was not specifically mentioned, but their bar graph suggests that in the left lateral hand area bodies and/or body parts are either preferred more than tools (and are thus the second preferred category) or are equally preferred to tools. This would correspond with our results. In the right lateral hand area, they found bodies to be preferred more than tools, again in accordance with our results. In 2016, Bracci and colleagues [13] then found that the response to bodies was higher than to tools, in all hand areas, supporting our findings, except for the lack of this effect in our study in the left ventral hand area. The reason for this lack of effect is unclear.

In the left and right lateral hand areas, we observed evidence that the tools were preferred over faces. In previous studies [10, 11, 13], faces were not included and therefore we cannot determine if our findings have been replicated before.

We also investigated the exclusive hand areas (i.e. not including the voxels overlapping with the tool areas). In the exclusive hand areas, the response to tools was significantly higher than to other objects (left lateral: *t*(18) = 2.98; *p* = .008; left ventral: *t*(18) = 2.98; *p* = .008; right lateral: *t*(18) = 6.63; *p* = 3.19*10–6; right ventral: *t*(14) = 4.18; *p* = 9.27*10–4). In the left lateral and the right lateral hand area (although only if *p* was uncorrected), the response to tools was also higher than to faces (left lateral: *t*(18) = 4.3; *p* = 4.33*10–4; left ventral: *t*(18) = -0.49; *p* = .63; right lateral: *t*(18) = 2.18; *p* = .04; right ventral: *t*(14) = 0.74; *p* = .47). In all hand areas (in left ventral only if *p* was uncorrected), the response to bodies was higher than to tools (left lateral: *t*(18) = -6.22; *p* = 7.21*10–6; left ventral: *t*(18) = -2.26; *p* = .04; right lateral: t(18) = -6.04; *p* = 1.04*10–5; right ventral: *t*(14) = -4.20; *p* = 8.86*10–4).

**Fig 7:**
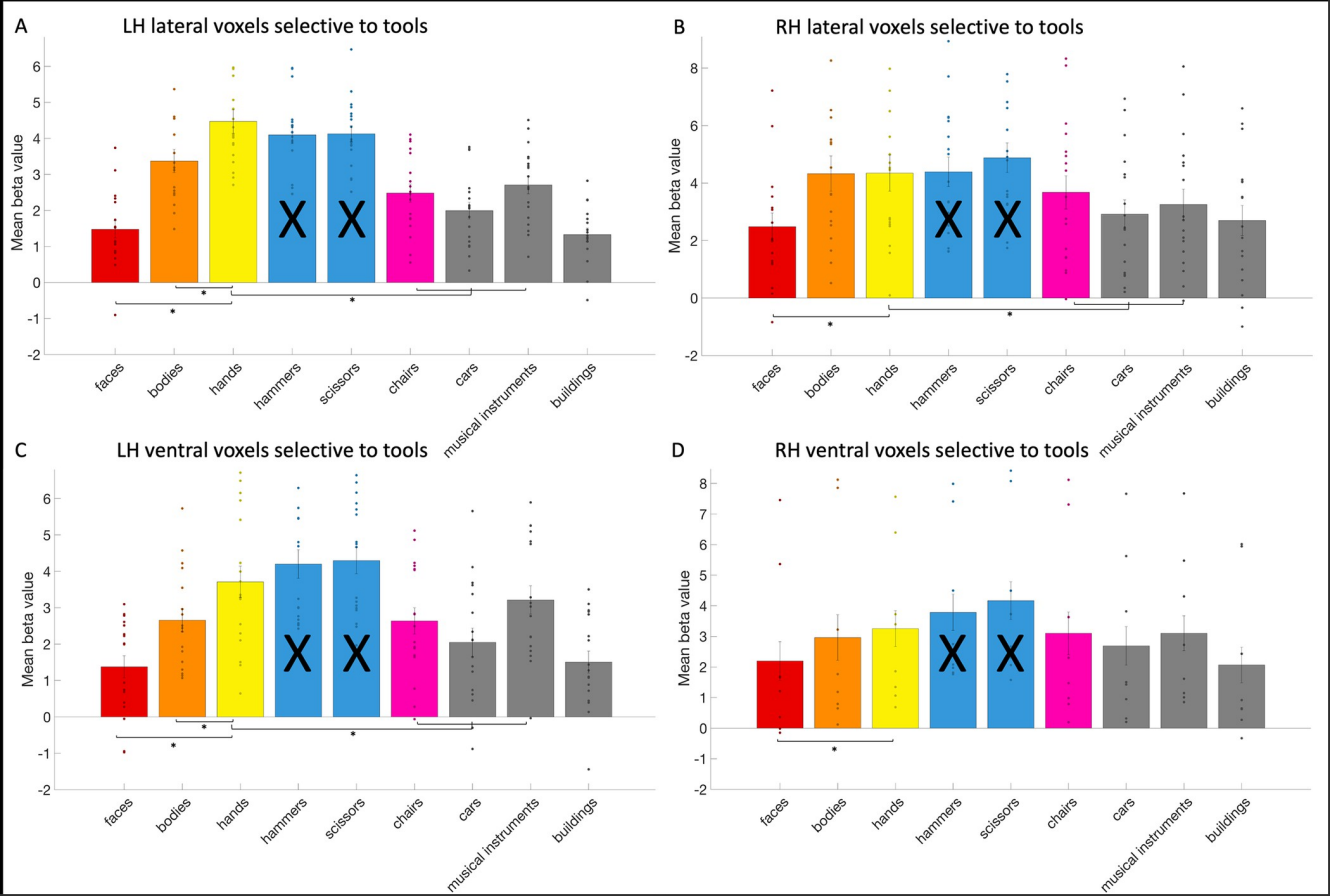
The response profile of the tool areas. The response profile on average and per subject (visualized by a dot per category bar) to faces, bodies, hands, hammers, scissors, chairs, cars, musical instruments, and buildings in the (A) left lateral (B) right lateral (C) left ventral and (D) right ventral tool areas. The results regarding to tools could not be interpreted (indicated by a black cross across the bar) as this was already used to define the tool areas. Significant statistical tests between categories were indicated using a *.

Next, we investigated hand selectivity in the tool areas. We could define the left lateral tool area (mean size: 34.32 voxels, standard deviation: 22.65) in 18 subjects, the left ventral tool area (mean size: 6.68 voxels, standard deviation: 8.73) in 17 subjects, the right lateral tool area (mean size: 13.53 voxels, standard deviation: 15.89) in 17 subjects, and the right ventral tool area (mean size: 2.68 voxels, standard deviation: 5.79) in 8 subjects. In their 2012 study, Bracci and colleagues [11] faced challenges in the right lateral tool area and only defined the left one. In their 2016 study [13] however, they could define right tool areas in most of their participants, as much as they could define right hand areas. The reason behind the absence of a tool area in the right ventral OTC remains unclear. In the left lateral tool area (see Fig 7), we found that the response to hands was significantly (i.e., *p* < .02 based on Bonferroni correction) higher than to faces (*t*(17) = 12.78; *p* = 3.82*10^−10^), bodies (*t*(17) = 6.94; *p* = 2.38*10^−6^) and objects (chairs, cars and musical instruments, *t*(17) = 8.78; *p* = 1.01*10^−7^). In the right lateral tool area, the response to hands and bodies (*t*(16) = 0.08; *p* = .94) did not differ significantly, but the response to hands was higher than to faces (*t*(16) = 5.06; *p* = .0001) and objects (*t*(16) = 3.4; *p* = .004). In the left ventral tool area, the response to hands was significantly higher than to faces (*t*(16) = 7.07; *p* = 2.58*10^−6^), bodies (*t*(16) = 4.7; *p* = .0002) and objects (*t*(16) = 3.65; *p* = .002). In the right ventral tool area, the response to hands was higher than to faces (*t*(7) = 4.95; *p* = .002), but not higher than bodies (*t*(7) = 0.85; *p* = .42) or objects (*t*(7) = 1.77; *p* = .12).

Thus, the left lateral and left ventral areas exhibited a preference for hands over the other categories (faces, bodies, objects that included chairs, cars and musical instruments). In right lateral and right ventral areas, hands were differentiated from faces and in the case of the right lateral area, also from objects, but not from bodies. These findings are more specific but overall consistent with what was reported by Bracci and colleagues (2012), study one [11]: they found that the left lateral tool area (they did not analyze other tool areas) exhibited a stronger response to hands than to animals or scenes. In study two, they also found hands to be the most preferred category, also more than bodies and body parts. A more recent study by Bracci and colleagues in 2016 [13], investigated all tool areas. They found the response to hands to be higher than to bodies and objects (they did not include faces) in the left lateral tool area, just like in their previous studies and our study. In the right lateral tool area, they found that the response to bodies was actually higher than to hands, but the response to hands was still higher than to objects. This is similar to our results, as the response to hands was not higher than to bodies, but still higher than objects. In the ventral left tool area, they found no higher response to hands than to bodies or objects. This is in contrast with our results because the response to hands was higher than to objects and bodies. In the ventral right tool area, they found that the response to bodies and objects was equally higher than to hands. We found that the response to hands was not higher than bodies or objects (only higher than faces). However, it should be taken into account that this area could not be identified in more than half of the subjects, which may have impacted the statistics.

We also investigated the exclusive tool areas (i.e. not including the voxels overlapping with the hand areas). In all exclusive tool areas, the response to hands was significantly higher than to faces (left lateral: *t*(17) = 6.31; *p* = 7.88*10–6; left ventral: *t*(14) = 6.40; *p* = 1.66*10–5; right lateral: *t*(14) = 4.24; *p* = 8.20*10–4; right ventral: *t*(7) = 5.12; *p* = .0014). In the exclusive left lateral and ventral tool areas, the response to hands was significantly higher than to bodies (left lateral: *t*(17) = 4.07; *p* = 8.02*10–4; left ventral: *t*(14) = 3.65; *p* = .003; right lateral: *t*(14) = -0.86; *p* = .41; right ventral: *t*(7) = 0.59; *p* = .58) and other objects (left lateral: *t*(17) = 7.37; *p* = 1.09*10–6; left ventral: *t*(14) = 2.54; *p* = .02; right lateral: *t*(14) = 1.55; *p* = .14; right ventral: *t*(7) = 0.37; *p* = .72).

**Fig 8:**
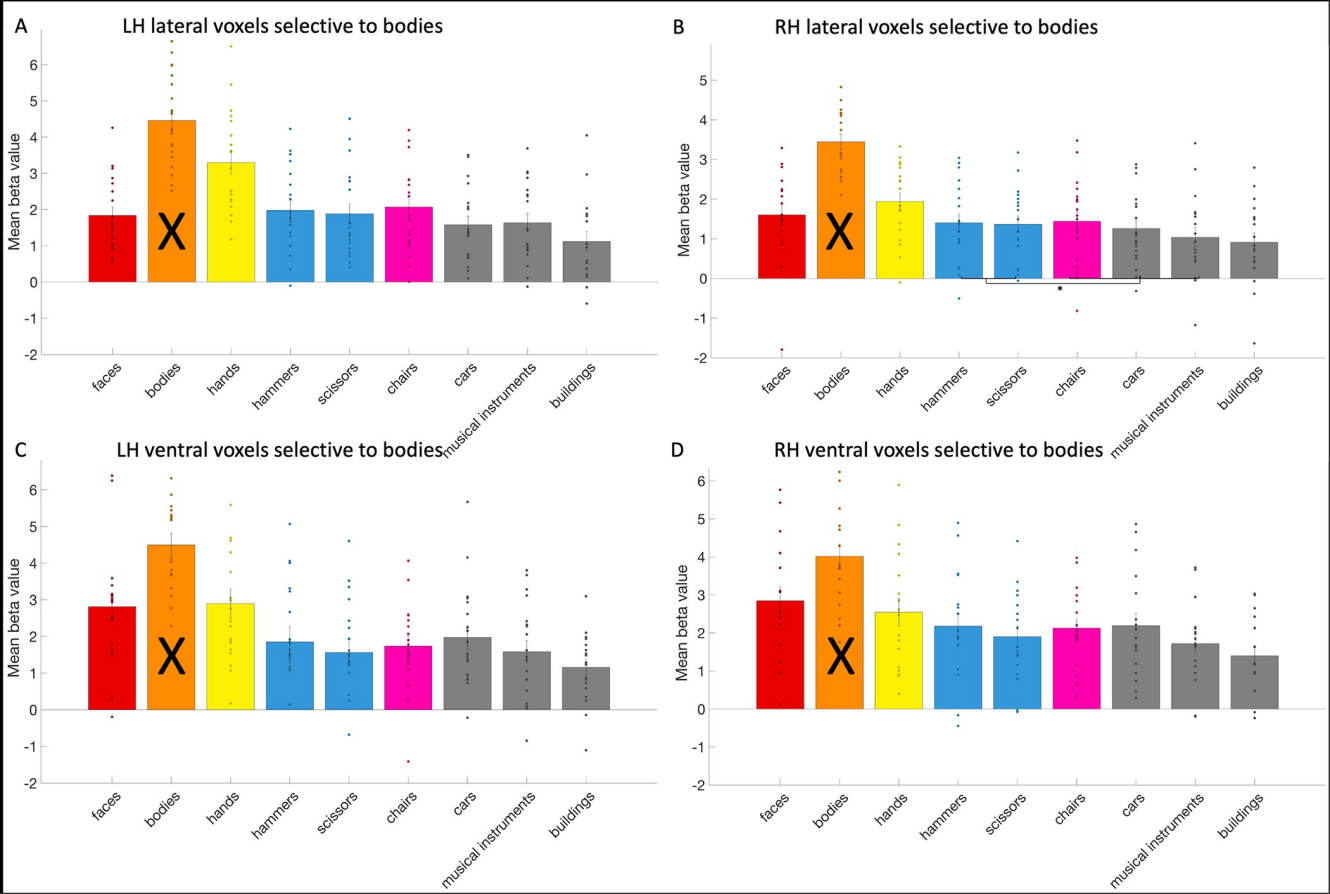
The response profile of the body areas. The response profile on average and per subject (visualized by a dot per category bar) to faces, bodies, hands, hammers, scissors, chairs, cars, musical instruments, and buildings in the (A) left lateral (B) right lateral (C) left ventral and (D) right ventral body areas. The results regarding to bodies could not be interpreted (indicated by a black cross across the bar) as this was already used to define the body areas. Significant statistical tests between categories were indicated using a *.

Next, we investigated tool selectivity in the body areas. We could define the left lateral (mean size: 56.42 voxels, standard deviation: 31.5), left ventral (mean size: 8.05 voxels, standard deviation: 5.7) and right lateral body area (mean size: 73 voxels, standard deviation: 45.68) in all 19 subjects and the right ventral body area (mean size: 10.63 voxels, standard deviation: 10.9) in 17 subjects. In the body areas (see Fig 8), as a control compared to the hand areas, we only examined whether these body areas could differentiate between tools and other objects. In the right lateral body area, the response to tools was significantly (i.e., *p* < .05) higher than to other objects (*t*(18) = 3.15; *p* = .006). In the left lateral, the left ventral and right ventral body areas, the difference between tools and objects was not significant (left lateral: *t*(18) = 0.87; *p* = .4; left ventral: *t*(18) = -0.28; *p* = .78; right ventral: *t*(16) = 0.21; *p* = .84).

Bracci and colleagues (2010) [10] demonstrated that body-selective voxels did not differentiate between tools and objects (chairs). In Bracci and colleagues (2012) study two [11], they also identified lateral body areas, but they did not mention testing if these areas differentiated between tools and objects (chairs). From their figure, it seems that the left lateral body area might have been able to (in contrast with our results), but the right lateral body area did not (also in contrast with our results as this was the one body area that we found that could make the differentiation).

**Fig 9:**
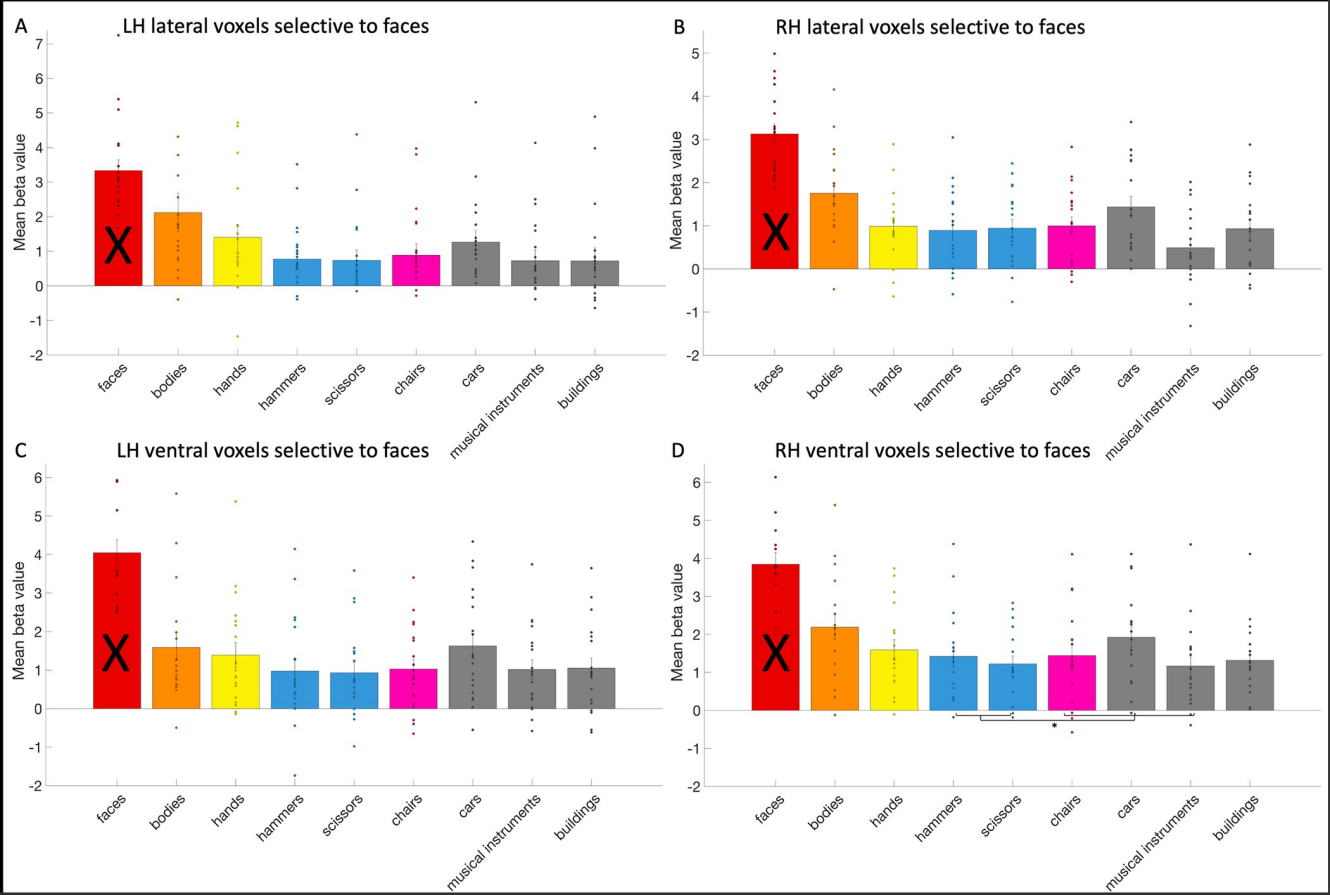
The response profile of the face areas. The response profile on average and per subject (visualized by a dot per category bar) to faces, bodies, hands, hammers, scissors, chairs, cars, musical instruments, and buildings in the (A) left lateral (B) right lateral (C) left ventral and (D) right ventral face areas. The results regarding to faces could not be interpreted (indicated by a black cross across the bar) as this was already used to define the face areas. Significant statistical tests between categories were indicated using a *.

In addition, we checked tool selectivity in the face areas. Previous studies did not include face areas for comparison [10, 11, 13]. We could define the left lateral (mean size: 37 voxels, standard deviation: 36.87) and ventral face area (mean size: 29.21 voxels, standard deviation: 21.15) in 19 subjects, the right lateral (mean size: 40.89 voxels, standard deviation: 39.63) in 18 subjects and the right ventral (mean size: 17.26 voxels, standard deviation: 12.85) in 17 subjects. Similar to the body areas, we examined if the face areas (see Fig 9), as a control for the hand areas, could differentiate between tools and other objects. In the right ventral face area, the response to objects was significantly (i.e., *p* < .05) higher than to tools (*t*(16) = -2.57; *p* = .02). In the left lateral, right lateral and left ventral face areas, the difference between tools and objects was not significant (left lateral: *t*(18) = -1.32; *p* = .2; right lateral: *t*(17) = -0.57; *p* = .58; left ventral: *t*(18) = -2.01; *p* = .06).

**Fig 10:**
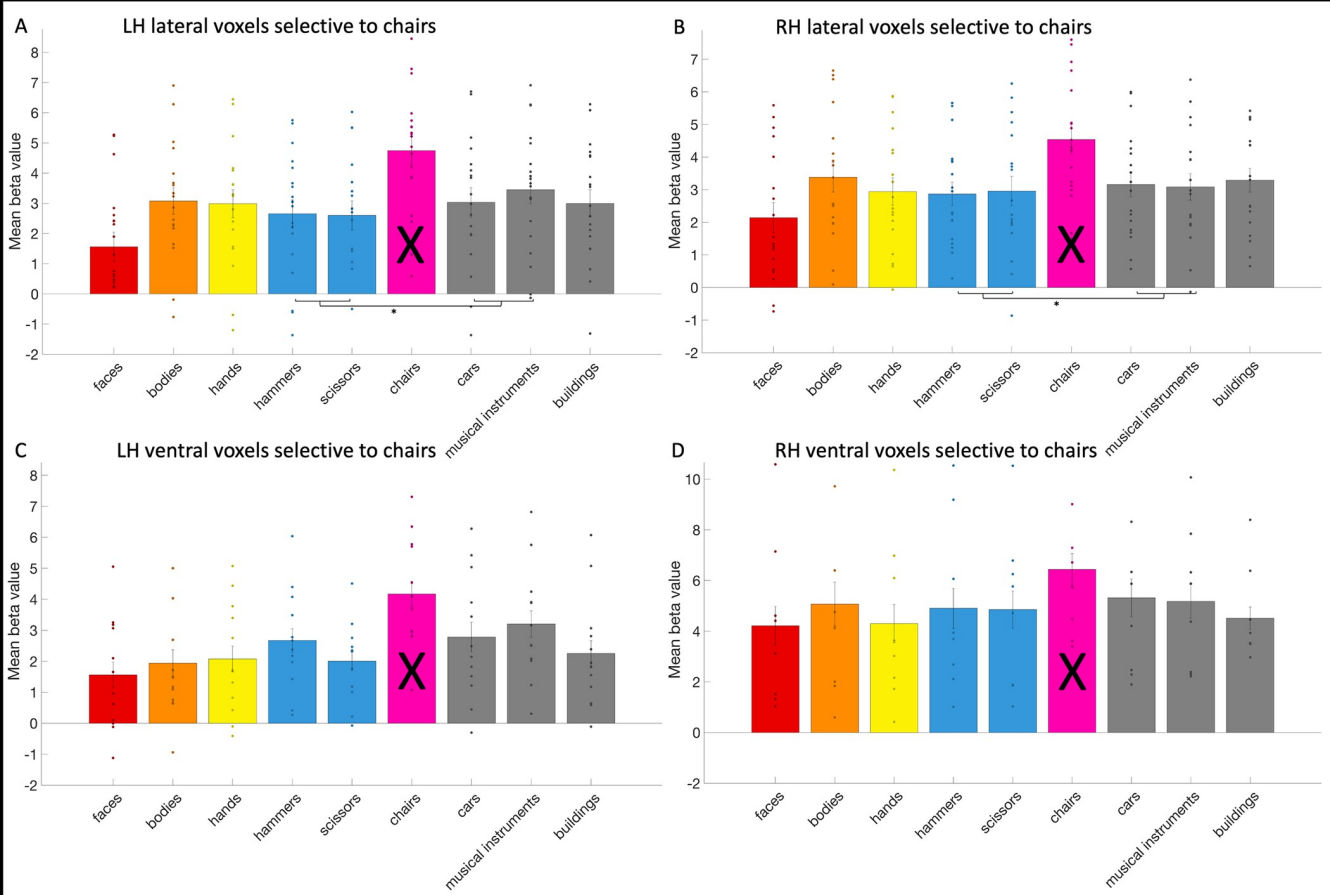
The response profile of the object (chair) areas. The response profile on average and per subject (visualized by a dot per category bar) to faces, bodies, hands, hammers, scissors, chairs, cars, musical instruments, and buildings in the (A) left lateral (B) right lateral (C) left ventral and (D) right ventral object/chair areas. The results regarding to the object chairs could not be interpreted (indicated by a black cross across the bar) as this was already used to define the object/chair areas. Significant statistical tests between categories were indicated using a *.

Finally, as another control, we identified object areas (see Fig 10) using the chairs category. We could define a left lateral object area (mean size: 34.21 voxels, standard deviation: 43.06) in 19 subjects, a left ventral (mean size: 1.47 voxels, standard deviation: 2.2) in 12 subjects, a right lateral (mean size: 28.32 voxels, standard deviation: 31.51) in 18 subjects and a right ventral (mean size: 1.47 voxels, standard deviation: 3.06) in 8 subjects. We then examined in each of them if they differentiated between tools and other objects (cars and musical instruments). Only one prior study defined a lateral occipital object region, but did not conduct a similar test in this area [11]. We found a differentiation between tools and other objects, where the response to objects was higher than to tools, in the left lateral and right lateral object areas (left lateral: *t*(18) = -2.92; *p* = .009; right lateral: *t*(17) = -2.72; *p* = .01). There was only a trend in left ventral object area (*t*(11) = -2.07; *p* = .06) and in the right ventral object area (*t*(7) = -2.1; *p* = .07).

### The representational space of hand and tool areas

**Fig 11:**
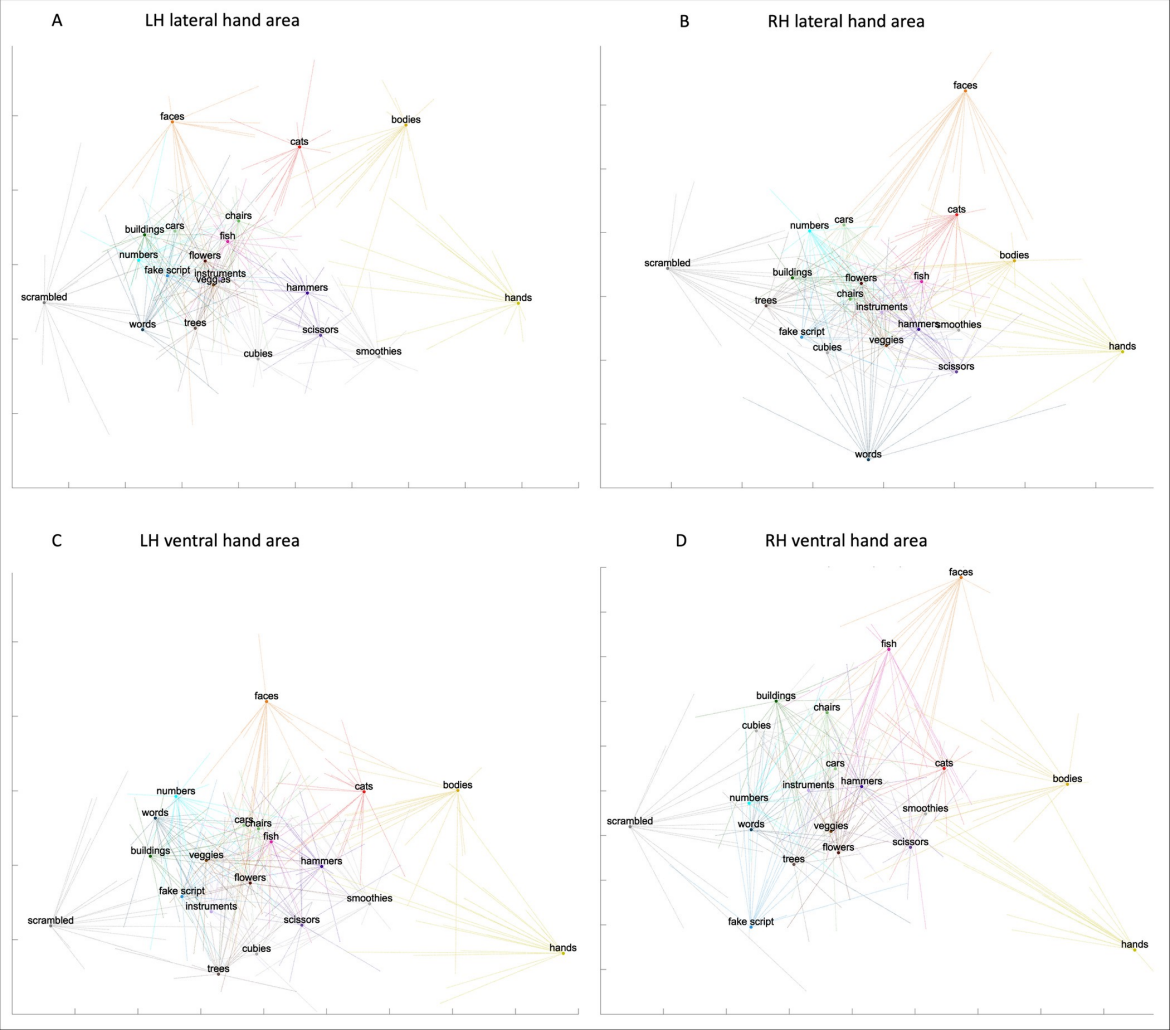
The representational space of the hand areas. Visualization of the average multi-voxel pattern analysis results in a two-dimensional space using MDS, for the (A) left lateral hand area, (B) right lateral hand area, (C) left ventral hand area, and the (D) right ventral hand area. Each participant’s MDS results were Procrustes transformed to the average MDS results and were shown by one line per category per subject in each of the four spaces.

**Fig 12:**
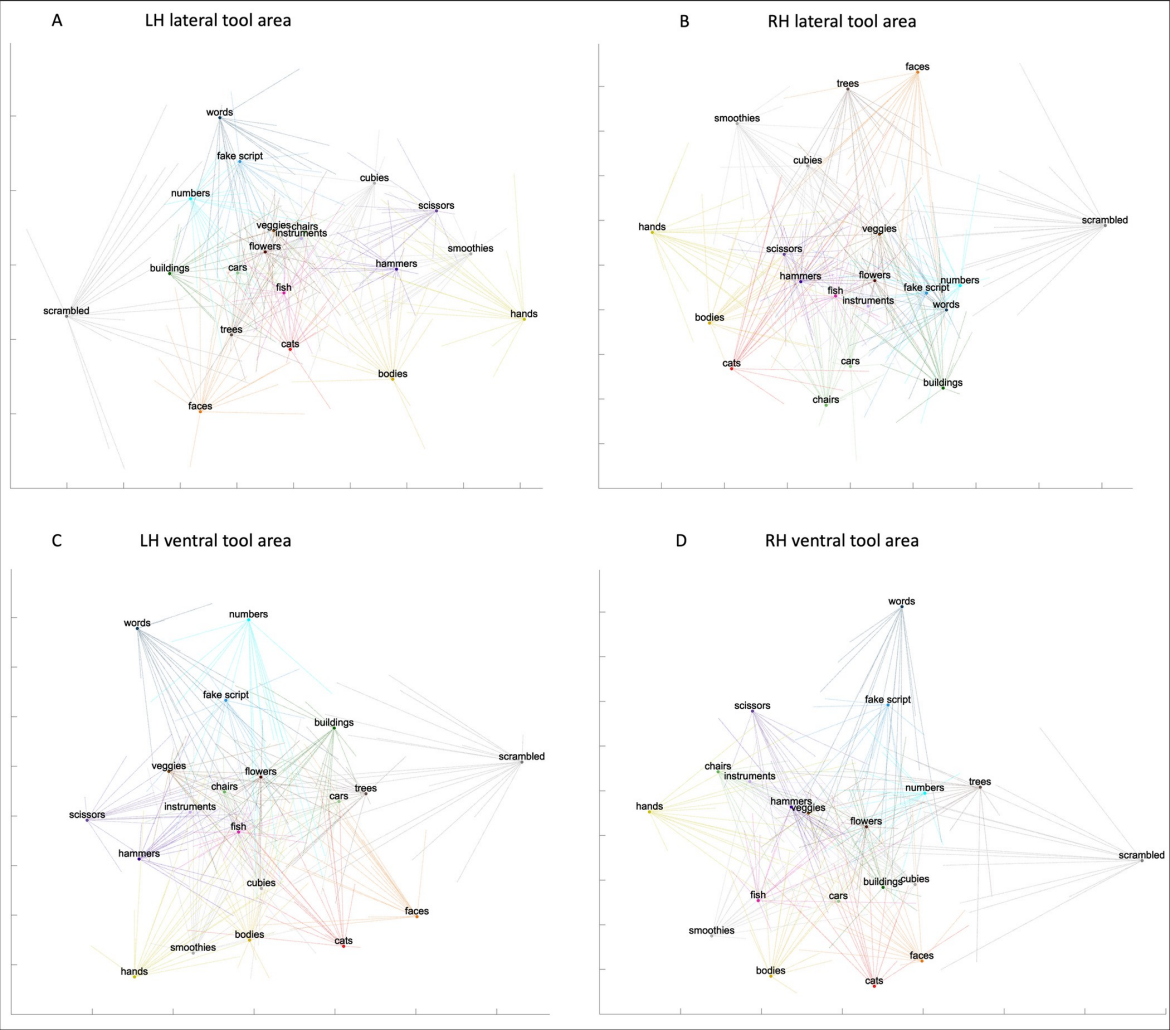
The representational space of the tool areas. Visualization of the average multi-voxel pattern analysis results in a two-dimensional space using MDS, for the (A) left lateral tool area, (B) right lateral tool area, (C) left ventral tool area, and the (D) right ventral tool area. Each participant’s MDS results were Procrustes transformed to the average MDS results and were shown by one line per category per subject in each of the four spaces.

We characterized the representational space in response to all categories of the hand and tool areas in left/right lateral/ventral OTC. Employing both univariate and multivariate analyses provides a multifaceted approach to examining the data, allowing for diverse perspectives. These complementary analyses can help understand the roles that these areas and their overlap might play in visual recognition and the action domain (see e.g., [12–14]). To achieve this, we defined the ROIs by intersecting the anatomical ROIs that we drew with the results of a GLM (hands vs all or tools vs all, except fixation) based on only half of the runs, for every subject. We then ran a multi-voxel pattern analysis using independent data (the GLM based on the other half of the runs), per subject. This resulted in a matrix per ROI and per subject, which were consequently normalized and then averaged across participants. To estimate reliability, each subject’s matrix was correlated to the average matrix. The mean correlation for the ROIs was as follows: left lateral hand area: *r* = .68, left ventral hand area: *r* = .45, right lateral hand area: *r* = .51, right ventral hand area: *r* = .42, left lateral tool area: *r* = .58, left ventral tool area: *r* = .35, right lateral tool area: *r* = .46, right ventral tool area: *r* = .29. We then visualized the MVPA results of every ROI in a two-dimensional space using MDS (see Fig 11 for the hand areas and Fig 12 for the tool areas). In this 2D space, categories that are close together exhibit more similar multi-voxel patterns (neural representations) than those that are farther apart. Within these 2D spaces, we also displayed the MDS results of every subject after Procrustes transformation: one line for every subject, per category and per space.

We first focused on results from the hand areas (see Fig 11). We defined (the size of the ROI needed to be larger than 1 voxel) a left lateral hand area in all 19 subjects (mean size: 125.16 voxels, standard deviation: 73.97), a left ventral in all 19 subjects (mean size: 23.37 voxels, standard deviation: 14.24), a right lateral in all 19 subjects (mean size: 78.58 voxels, standard deviation: 86.07) and a right ventral (mean size: 9.21 voxels, standard deviation: 8.02) in 15 subjects. Our main interest, as in the univariate analysis, was the potential differentiation between tools and other objects. For comparison, we selected chairs, cars, and musical instruments as typical objects. In each hand area, we tested (using a paired t-test), whether the distance as noted in the dissimilarity matrix (the normalized LDC value of each subject) of hands–hammers and hands–scissors was smaller than the distance between hands–chairs, hands–cars, ands hands–musical instruments. Indeed, in all hand areas except the right ventral hand area (although it did show a trend), the distance was significantly (i.e., *p* < .01 based on Bonferroni correction) smaller (left lateral: *t*(18) = -11.26, *p* = 1.39*10^−9^; left ventral: *t*(18) = -3.8, *p* = .001; right lateral: *t*(18) = -9.82, *p* = 1.19*10^−8^; right ventral: *t*(14) = -2.12, *p* = .05). This indicated that the neural representations of hammers and scissors were more similar to that of hands than other typical object categories, in the hand areas.

Second, we investigated the results from the tool areas (see Fig 12). We defined a left lateral tool area in 18 subjects (mean size: 65.25 voxels, standard deviation: 37.7), a left ventral in 18 subjects (mean size: 18.42 voxels, standard deviation: 14.78), a right lateral in all 19 subjects (mean size: 82.16 voxels, standard deviation: 140.95) and a right ventral in 15 subjects (mean size: 13.37 voxels, standard deviation: 25.11). We tested whether the distance between tools and hands was significantly (i.e., *p* < .01 based on Bonferroni correction) smaller than between tools (including hammers and scissors) and objects (including chairs, cars and musical instruments). However, this was not the case (left lateral tool area: *t*(17) = -1.75, *p* = .1; left ventral: *t*(17) = -0.47, *p* = .65; right lateral: *t*(18) = 1.05, *p* = .31; right ventral: *t*(14) = -0.79, *p* = .45). Thus, the neural representations of hands and typical object categories were equally similar to the neural representation of tools in the tool areas. Bracci and colleagues (2012, 2016) [11, 13] also performed MVPA, but we cannot compare results because they conducted MVPA only in a general lateral OTC and not in hand and tool areas separately, or, they did not use methods/tests like ours.

## Discussion

Prior research on the hand and tool areas and the overlap between them in OTC suffers from several drawbacks that could have influenced their outcomes, such as the use of spatial normalizing and smoothing. To overcome these drawbacks, in this study, we reinvestigated these areas and their overlap in 19 participants, by omitting these preprocessing steps, by including a broad range of categories to establish a more comprehensive framework and by employing a modestly increased spatial resolution, made possible by the high signal-to-noise ratio that 7T fMRI provides. We examined the overlap in all parts of OTC. In addition, we explored the response profile of the hand and tool areas and compared to body, face and object (chair) areas. Then, we constructed the representational space of the hand and tool areas using multi-voxel pattern analysis.

First, overlap between areas was analyzed. Many studies demonstrated an overlap between the hand and tool selectivity and investigated why this overlap might exist [e.g., 11–14, 25–28, 31]. In our study, we demonstrated the existence of this overlap in the different parts of OTC, which is striking given that we scanned at a high field strength (7T), allowing to omit spatial preprocessing steps that can lead to artificial overlap. The overlap between hands and tools was specific in the left hemisphere, replicating prior research on left lateral OTC (they did not investigate beyond this region) [11]. It was less or not specific in the right hemisphere. Also, the overlap was not larger in lateral versus ventral OTC, not in the left or the right hemisphere. There was more overlap between in the left lateral OTC compared to the right lateral OTC, but not for the left versus right ventral OTC. Overall, these findings, specifically the trend that the overlap is more specific and larger in the left (lateral) hemisphere, may support the idea of a left-lateralized network for visual and motor control [63] and hand and tool action observation and execution [11]. This may also align with the concept of a left-lateralized organization where lateral OTC serves as a bridge between parietal regions and ventral OTC [13].

Second, the hand areas were identified, in line with prior studies [e.g., 11, 12, 14, 25–28]. The existence of hand areas beyond left lateral OTC is less well documented, but replicates another recent study [13, 32]. All four hands areas, not just the left lateral [10, 11, 13] made a distinction between tools and other typical objects, suggesting tools are meaningful to these areas. This finding cannot be explained by the important organizing principle of animacy [29], as tools are inanimate just like other types of objects. Some other connection between hands and tools may explain this, likely residing within the action domain given that hands and tools are not visually similar either [11, 12, 14, 25, 27, 28]. Another potential explanation is rooted in the statistics of the visual input in which hands and tools often occur together. The hand areas overall also responded more to bodies than to tools (also see [11, 13]). Hands and bodies are both animate categories and are intrinsically linked, supporting the idea of both animacy and body topography as organizing principles of OTC [29, 64]. In the lateral hand areas, the response to tools was stronger than to faces and in the ventral hand areas, there was no difference. This is surprising given that faces are animate (like hands and bodies), but the response to tools (inanimate,) was stronger than or equal to that of faces. These findings are further evidence for a functional connection beyond animacy or visual similarity between hands and tools, a connection that is so strong that faces (but not bodies) are responded to less than tools. We also investigated the exclusive hand areas excluding overlapping voxels with the tool areas. All showed a significantly higher response to tools than to non-tool objects indicating that there are no exclusively selective hand areas if compared to tools, supporting the idea that hands and tools are intrinsically functionally connected in some way and that this shared information is being processed in the hand and tool areas.

Third, the tool areas were identified, in line with prior research [e.g., 13, 15–22]. Based on animacy, one might anticipate that these tool areas would favor objects over hands, bodies or faces, all of which are animate categories. In contrast, these tool areas preferred hands specifically to all other categories of interest (faces, bodies, objects). This effect was less pronounced in the right hemisphere tool areas. These findings (along with [11, 13]) lead to a similar conclusion as the findings from the hand areas: hands and tools share a special connection in some way that cannot be explained solely by the typical animacy dimension or visual similarity. We also investigated the exclusive hand and tool areas excluding overlapping voxels. All hand areas showed a significantly higher response to tools than to non-tool objects, and all tool areas showed a higher response to hands than to faces. This indicates that there are no exclusively selective hand/tool areas if compared to tools/hands, supporting the idea that hands and tools are intrinsically functionally connected in some way and that this shared information is being processed in the hand and tool areas.

Then, face, body and object areas were also identified as a control. These are located close to the hand and tool areas in OTC and are relevant semantically as well, for example based on the dimension of animacy [1–4, 29]. Only the body areas were tested similarly in prior studies, yielding mixed findings [10, 11]. Unlike the hand areas, most face and body areas could not differentiate between tools and other objects, which can be explained by animacy. It suggests that differentiating between tools and other objects is significant only for the hand areas, a phenomenon not explicable by animacy. The identified object (chair) areas overall preferred other typical objects more than it did tools. As both are inanimate categories, this finding is unexpected. This suggests that object regions process specific information not shared with tools.

Altogether, these findings suggest that animacy is one dimension determining the functional organization of OTC, but it likely interacts with other factors to explain the intricate landscape of OTC. In the case of the hand and tool areas and their overlap, findings suggest the action domain, where the two categories are functionally related, and in which other typical objects are distinguished from tools. Hands are often used to manipulate tools, that extend the body to cause certain effects. This combination of causality and body extension [14] can explain why hand areas and tool areas respond strongly to tools and hands, respectively. It could also explain why these areas cluster together and partially overlap [14]. However, other factors that have been previously proposed could also be used the explain the findings of this study [for more discussion, see 14, 25–28]. This study does not include the necessary complex stimuli/tasks to draw conclusions about which of the action-domain theories is accurate. Furthermore, to date, findings remain mixed, preventing a definitive conclusion [e.g., see 65 for evidence that may not support the causality and body extension account and also see 66].

Regardless of the exact factor(s) explaining the connection between hands and tools, it is evident from this line of studies that the functional organization of OTC is complex, going beyond simple category selectivity. This organization must efficiently process several (visual) features concurrently when presented with complex visual stimuli. This requires an intricate interplay between different visual areas within OTC. Additional examples supporting this conclusion, apart from the hand and tool areas and their overlap, include the separate but partially overlapping areas identified for faces and bodies [35], as well as the multiple limb-selective areas surrounding the motion-selective areas [33].

In addition, we investigated the representational space of the hand and tool areas. Within the hand areas, the representation of tools was more similar than that of objects, to the representation of hands. This aligns with the response profiles of the hand areas, where tools yielded a stronger response than other objects, providing further support for a special connection between hands and tools that cannot be explained by animacy. In the tool areas, we observed that the representations to hands and to typical objects were all equally similar to the representation of tools. One might have expected tools and hands to exhibit a similar neural representation based on previous studies, where there was no difference in response (or even a higher response) to hands versus tools in a univariate analysis of the tool areas [10, 11, 13]. Although due to methodological differences in the MVPA, we cannot compare to prior research by Bracci and colleagues [11, 13], these authors did propose an interesting account of the different roles that these areas might play [13]. They proposed that the left lateral OTC serves as a bridge between the areas in the left parietal cortex and left ventral OTC, that respond to hands and/or tools [13]. Conducting functional connectivity research between these areas is a next crucial step to validate this theory [13].

It is noteworthy that, especially in the univariate analyses, certain category-selective areas could not be identified in a substantial number of participants (e.g., the right ventral tool area), impacting the statistics in these regions (such as tests on its response profile and on its overlap with other areas). Another limitation of this study is that, during the creation of the anatomical-functional ROIs a bias was introduced, by using the results from relevant contrasts (e.g., hands vs all to create the left/right lateral/ventral hand areas). Consequently, the results from the category used in the contrast to define the ROI (e.g. the hands category in the case of the four hand areas), could not be interpreted.

A benefit of this study was the careful design of our stimuli, ensuring that bodies did not contain hands, similar to how they do not contain faces, as these categories are intended to be distinct. While we do not claim that this detail influenced previous studies, we believe it could be a valuable practice for future research, as its impact may not be immediately apparent. Similarly, segmenting bodies into distinct parts may be pertinent for future investigations, particularly given a recent study’s findings suggesting that in ventral OTC, representations of the whole body cluster together with those of body parts, indicating that selectivity for bodies may, in part, reflect selectivity for body parts [67]. Another rationale for subdividing bodies into parts, especially into a limbs category, when studying hands and tools, is that hands and limbs are similarly represented in OTC due to their common role as action effectors [68].

Another important benefit of this study was the use of 7T fMRI, which yielded high-quality data due to an enhanced signal-to-noise ratio [36]. This enabled the incorporation of a diverse range of categories, thereby facilitating a more comprehensive understanding of the hand and tool areas and their overlap. In addition, we could emit spatial normalization and smoothing of the data. This was particularly crucial for analyzing the overlap between hand and tool areas, as smoothing might inadvertently blend regions that are anatomically distinct [35]. 7T fMRI also enabled the use of a modestly boosted spatial resolution [36], particularly beneficial for examining the potential overlap between hand and tool areas, which could otherwise be a result of insufficient spatial resolution [11]. However, given the inclusion of a diverse array of categories, the spatial resolution was only increased a little compared to previous studies. This leaves certain questions unanswered. For example, while our study suggests that overlap is not a consequence of specific spatial preprocessing steps, it remains possible that it could appear artificial when examined at an even higher spatial resolution.

Overall, these modifications due to 7T fMRI allowed us to corroborate several previous findings and expanded upon them in various ways. We showed that not only the left lateral, but all hand areas distinguished between tools and other objects. We also compared the tool responses not only with hand responses but also with several other human categories (bodies and faces). We confirmed that bodies were preferred over tools by the hand areas. In contrast, we revealed for the first time that hand areas responded more strongly to tools than to faces. We also demonstrated that not only the left lateral, but all tool areas distinguished between hands, bodies in the left hemisphere, faces and other types of objects than tools. To our knowledge, our study is the first to investigate the overlap between hand and tool areas beyond the left lateral OTC [11]. The robust finding of overlap in OTC is striking given we scanned at 7T, allowing the omission of spatial preprocessing steps that could have led to artificial overlap in prior research. The overlap was specific to hands and tools in the left hemisphere, and less so in the right hemisphere. The size of the overlap did not significantly differ between lateral and ventral OTC (per hemisphere), but it was larger in the left versus right lateral OTC. When excluding the overlapping voxels from the hand and tool areas, all the areas still showed a significant response to tools or hands respectively. Then, we constructed the representational space of all hand and tool areas and compared the distance between neural representations of several category pairs, a step not undertaken in previous studies. Overall, these findings indicate that on top of the well-known animacy dimension, other features also play a role in determining the functional organization of OTC, and in the case of the hand and tool areas, evidence supports a functional connection between these categories in the action domain.

Future work could further investigate the distinct roles of these various hand and tool areas. Although we used a modestly higher spatial resolution than previous studies and demonstrated overlap between hand and tool areas, despite omitting spatial preprocessing steps that might have influenced this overlap, it remains possible that an even higher spatial resolution would reveal these areas as separate and positioned really close to one another, or that there is no overlap at the level of single-neuron selectivity. Additionally, conducting functional connectivity studies between these four hand and tool areas with each other and other regions (e.g., action-related regions) would enrich the field’s understanding of the roles of these areas in OTC and beyond. More in depth research into the anatomical position of these hand and tool areas and their relative position to one another would also be valuable to future investigations into these areas. As a last suggestion, as described in detail above, future studies could include several body part categories, especially a limbs category, when studying hands and tools. This is especially relevant considering that in OTC, there are representations not only of whole bodies but also of body parts, and given the similarity in the neural representations of hands and limbs as action effectors [67, 68].

## Supporting information

S1 Text Supplementary methods. A detailed description of the preprocessing steps, provided by fMRIPrep.
(DOCX)

S1 Fig Hand, tool and object areas and their overlap. Hand, tool and object areas and their overlap, determined by a contrast of one versus all other categories except fixation, p < 0.05, FWE corrected (hands in yellow, tools in blue, objects (chairs) in pink, overlap between hands and tools in green, between hands and objects in brown, between tools and objects in purple and between hands, tools and objects in white), shown upon annotated left lateral (top left), posterior (top right), right lateral (bottom left) and ventral (bottom right) brain surface of participant 11 (color legend also on the right of the figure).
(TIF)

S2 Fig Hand, face, body and tool areas and their overlap. Hand, face, body and tool areas and their overlap, determined by a contrast of one versus all other categories except fixation, p < 0.05, FWE corrected (hands in yellow, faces in red, bodies in orange, overlap between some or all of these three animate categories in brown, tools in blue, overlap between hands and tools in green, and between animate categories and tools in white), shown upon annotated left lateral (top left), posterior (top right), right lateral (bottom left) and ventral (bottom right) brain surface of participant 11 (color legend also on the right of the figure).
(TIF)

S3 Fig Hand, tool and object areas and their overlap. Hand, tool and object areas and their overlap, determined by a contrast of one versus all other categories except fixation, p < 0.05, FWE corrected (hands in yellow, tools in blue, objects (chairs) in pink, overlap between hands and tools in green, between hands and objects in brown, between tools and objects in purple and between hands, tools and objects in white), shown upon annotated left lateral (top left), posterior (top right), right lateral (bottom left) and ventral (bottom right) brain surface of participant 8 (color legend also on the right of the figure).
(TIF)

S4 Fig Hand, face, body and tool areas and their overlap. Hand, face, body and tool areas and their overlap, determined by a contrast of one versus all other categories except fixation, p < 0.05, FWE corrected (hands in yellow, faces in red, bodies in orange, overlap between some or all of these three animate categories in brown, tools in blue, overlap between hands and tools in green, and between animate categories and tools in white), shown upon annotated left lateral (top left), posterior (top right), right lateral (bottom left) and ventral (bottom right) brain surface of participant 8 (color legend also on the right of the figure).
(TIF)

S5 Fig Hand, tool and object areas and their overlap. Hand, tool and object areas and their overlap, determined by a contrast of one versus all other categories except fixation, p < 0.05, FWE corrected (hands in yellow, tools in blue, objects (chairs) in pink, overlap between hands and tools in green, between hands and objects in brown, between tools and objects in purple and between hands, tools and objects in white), shown upon annotated left lateral (top left), posterior (top right), right lateral (bottom left) and ventral (bottom right) brain surface of participant 17 (color legend also on the right of the figure).
(TIF)

S6 Fig Hand, face, body and tool areas and their overlap. Hand, face, body and tool areas and their overlap, determined by a contrast of one versus all other categories except fixation, p < 0.05, FWE corrected (hands in yellow, faces in red, bodies in orange, overlap between some or all of these three animate categories in brown, tools in blue, overlap between hands and tools in green, and between animate categories and tools in white), shown upon annotated left lateral (top left), posterior (top right), right lateral (bottom left) and ventral (bottom right) brain surface of participant 17 (color legend also on the right of the figure).
(TIF)

S7 Fig Hand, tool and object areas and their overlap. Hand, tool and object areas and their overlap, determined by a contrast of one versus all other categories except fixation, p < 0.05, FWE corrected (hands in yellow, tools in blue, objects (chairs) in pink, overlap between hands and tools in green, between hands and objects in brown, between tools and objects in purple and between hands, tools and objects in white), shown upon annotated left lateral (top left), posterior (top right), right lateral (bottom left) and ventral (bottom right) brain surface of participant 18 (color legend also on the right of the figure).
(TIF)

S8 Fig Hand, face, body and tool areas and their overlap. Hand, face, body and tool areas and their overlap, determined by a contrast of one versus all other categories except fixation, p < 0.05, FWE corrected (hands in yellow, faces in red, bodies in orange, overlap between some or all of these three animate categories in brown, tools in blue, overlap between hands and tools in green, and between animate categories and tools in white), shown upon annotated left lateral (top left), posterior (top right), right lateral (bottom left) and ventral (bottom right) brain surface of participant 18 (color legend also on the right of the figure).
(TIF)
