# Supplementary figures and images for: A 7T fMRI investigation of hand and tool areas in the lateral and ventral occipitotemporal cortex
Source: PLoS One. 2024 Nov 5;19(11):e0308565. doi: 10.1371/journal.pone.0308565 (PMC11537398; doi:10.1371/journal.pone.0308565)

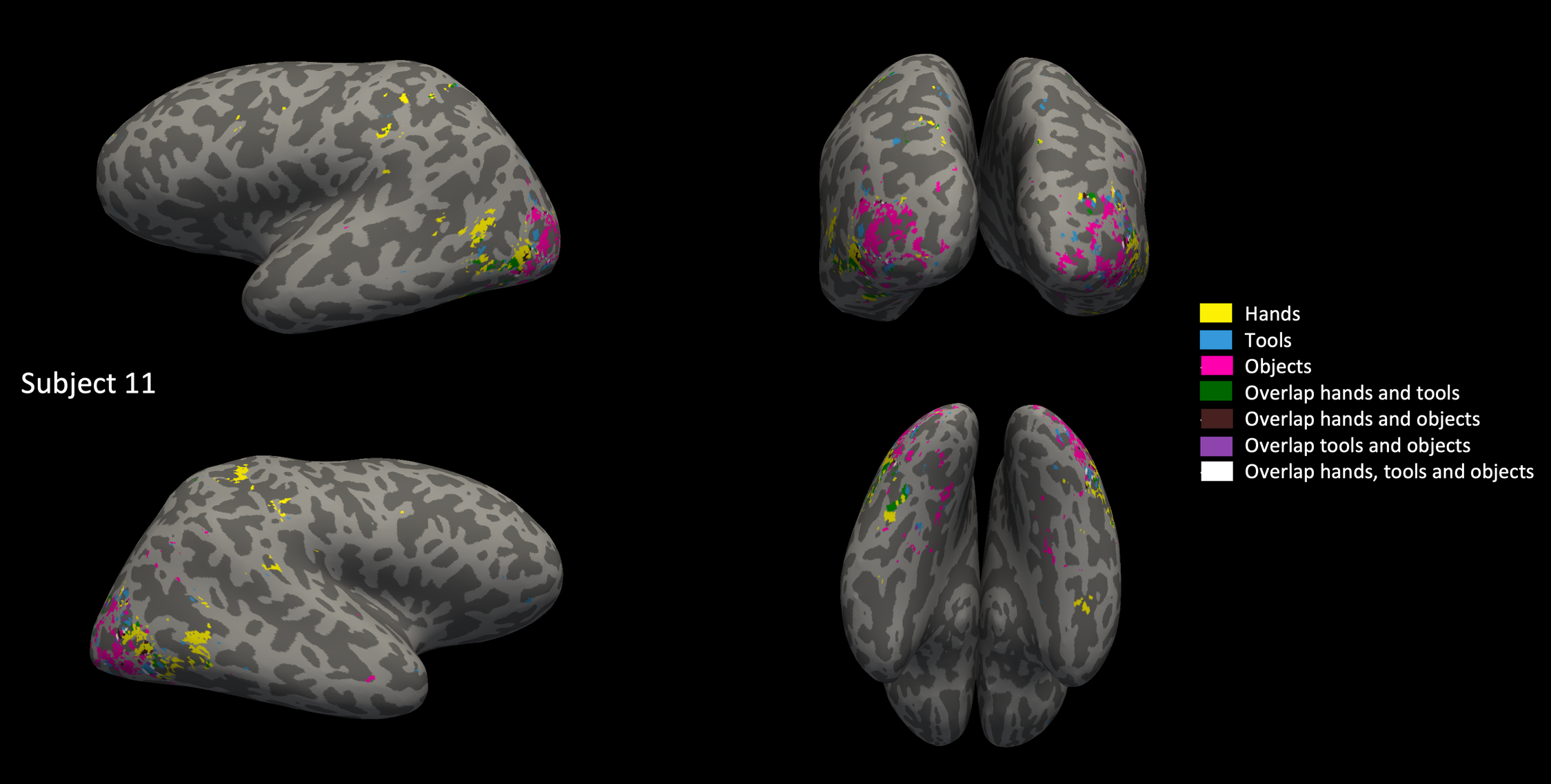

Supplement: S1 Fig — Hand, tool and object areas and their overlap, determined by a contrast of one versus all other categories except fixation, p < 0.05, FWE corrected (hands in yellow, tools in blue, objects (chairs) in pink, overlap between hands and tools in green, between hands and objects in brown, between tools and objects in purple and between hands, tools and objects in white), shown upon annotated left lateral (top left), posterior (top right), right lateral (bottom left) and ventral (bottom right) brain surface of participant 11 (color legend also on the right of the figure). (TIF) [file pone.0308565.s002.tif]

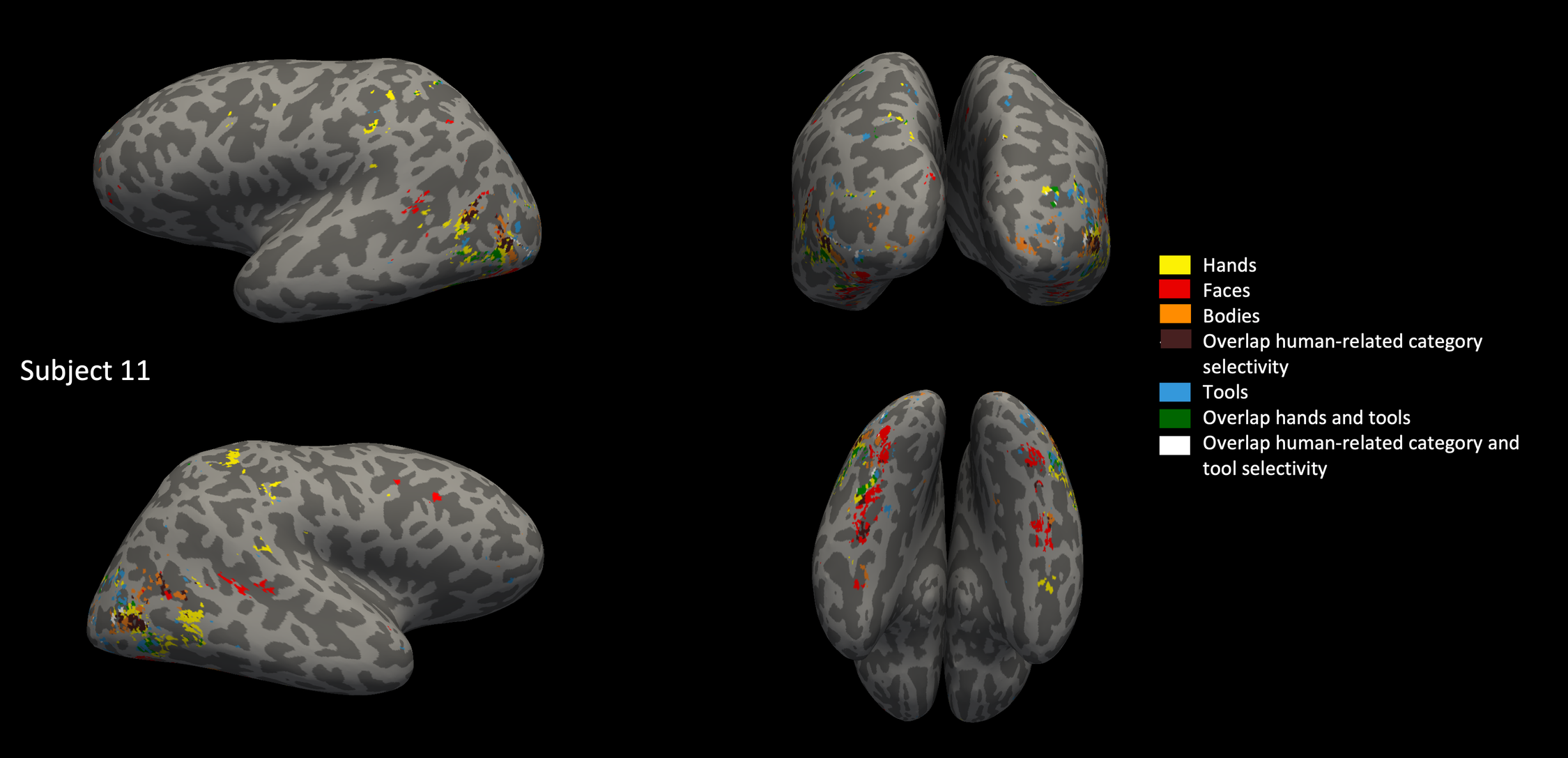

Supplement: S2 Fig — Hand, face, body and tool areas and their overlap, determined by a contrast of one versus all other categories except fixation, p < 0.05, FWE corrected (hands in yellow, faces in red, bodies in orange, overlap between some or all of these three animate categories in brown, tools in blue, overlap between hands and tools in green, and between animate categories and tools in white), shown upon annotated left lateral (top left), posterior (top right), right lateral (bottom left) and ventral (bottom right) brain surface of participant 11 (color legend also on the right of the figure). (TIF) [file pone.0308565.s003.tif]

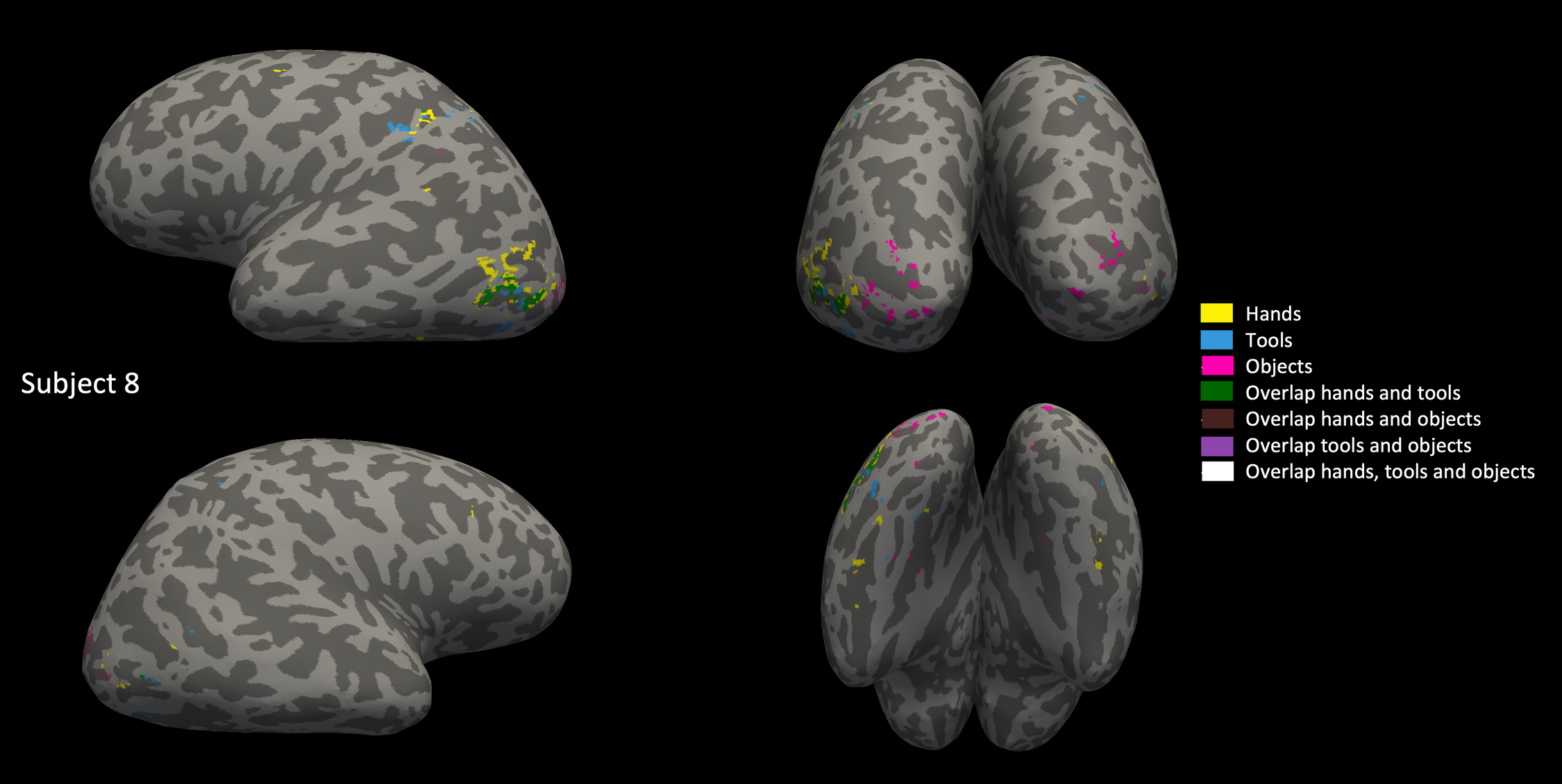

Supplement: S3 Fig — Hand, tool and object areas and their overlap, determined by a contrast of one versus all other categories except fixation, p < 0.05, FWE corrected (hands in yellow, tools in blue, objects (chairs) in pink, overlap between hands and tools in green, between hands and objects in brown, between tools and objects in purple and between hands, tools and objects in white), shown upon annotated left lateral (top left), posterior (top right), right lateral (bottom left) and ventral (bottom right) brain surface of participant 8 (color legend also on the right of the figure). (TIF) [file pone.0308565.s004.tif]

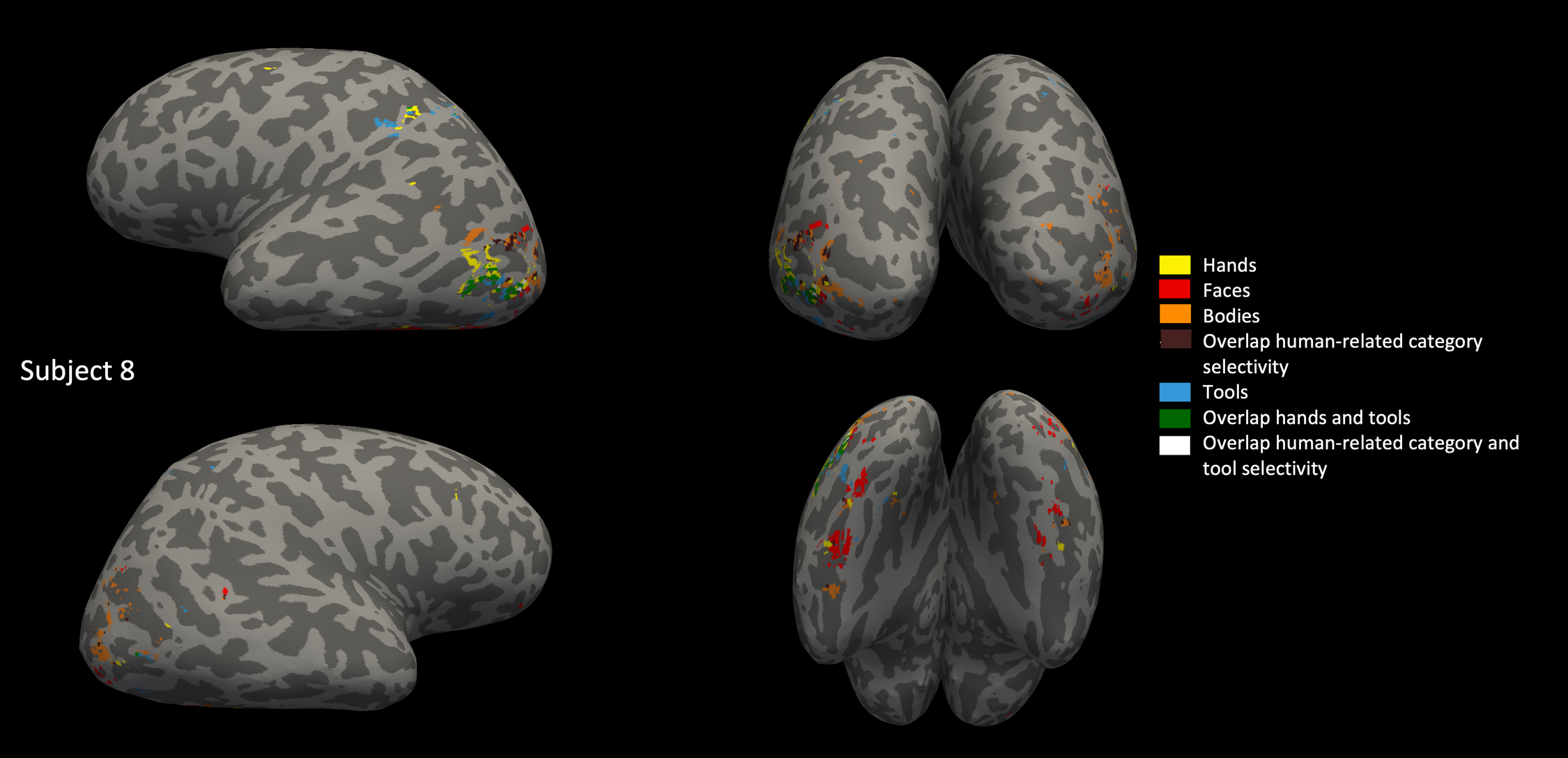

Supplement: S4 Fig — Hand, face, body and tool areas and their overlap, determined by a contrast of one versus all other categories except fixation, p < 0.05, FWE corrected (hands in yellow, faces in red, bodies in orange, overlap between some or all of these three animate categories in brown, tools in blue, overlap between hands and tools in green, and between animate categories and tools in white), shown upon annotated left lateral (top left), posterior (top right), right lateral (bottom left) and ventral (bottom right) brain surface of participant 8 (color legend also on the right of the figure). (TIF) [file pone.0308565.s005.tif]

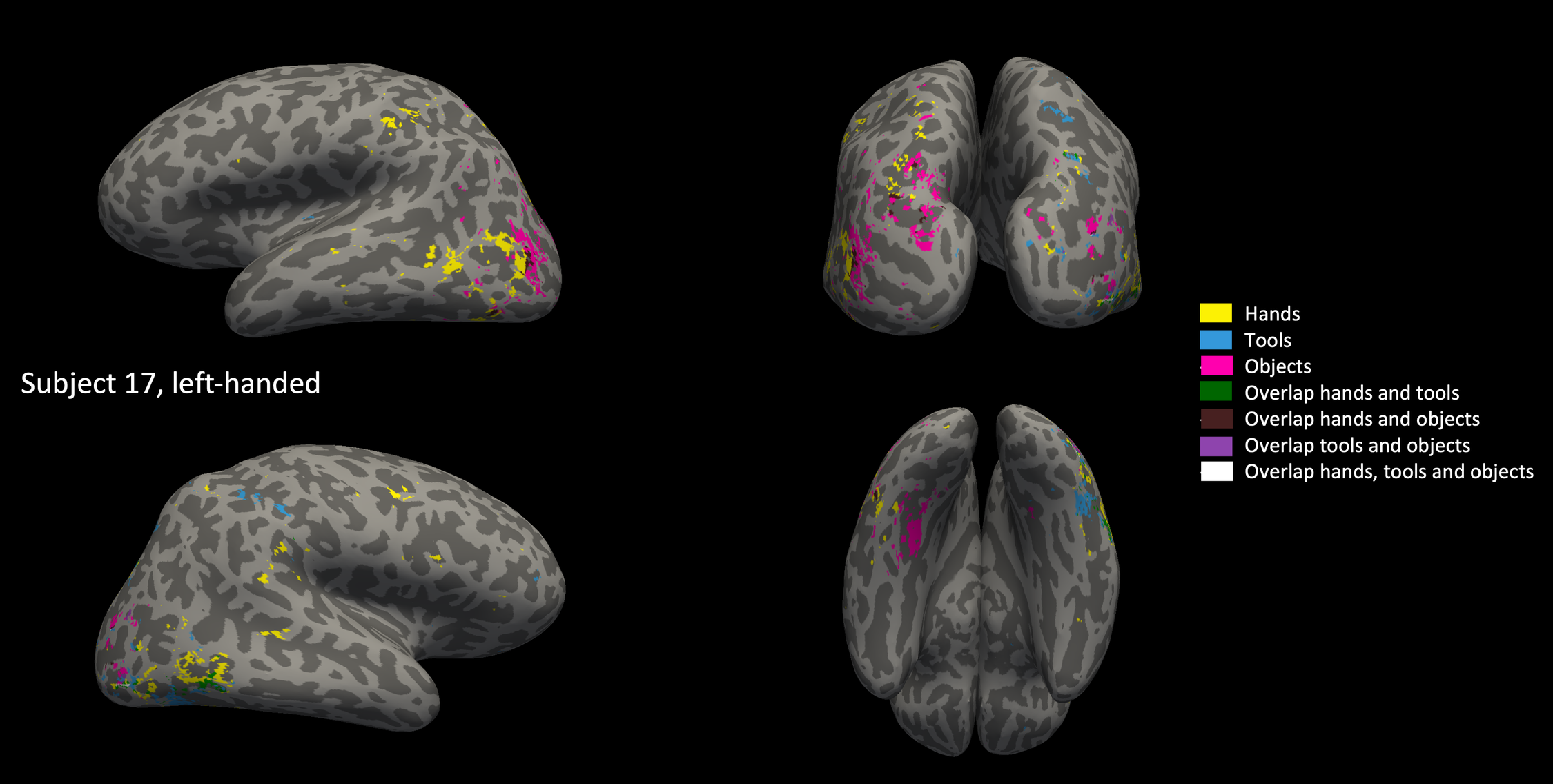

Supplement: S5 Fig — Hand, tool and object areas and their overlap, determined by a contrast of one versus all other categories except fixation, p < 0.05, FWE corrected (hands in yellow, tools in blue, objects (chairs) in pink, overlap between hands and tools in green, between hands and objects in brown, between tools and objects in purple and between hands, tools and objects in white), shown upon annotated left lateral (top left), posterior (top right), right lateral (bottom left) and ventral (bottom right) brain surface of participant 17 (color legend also on the right of the figure). (TIF) [file pone.0308565.s006.tif]

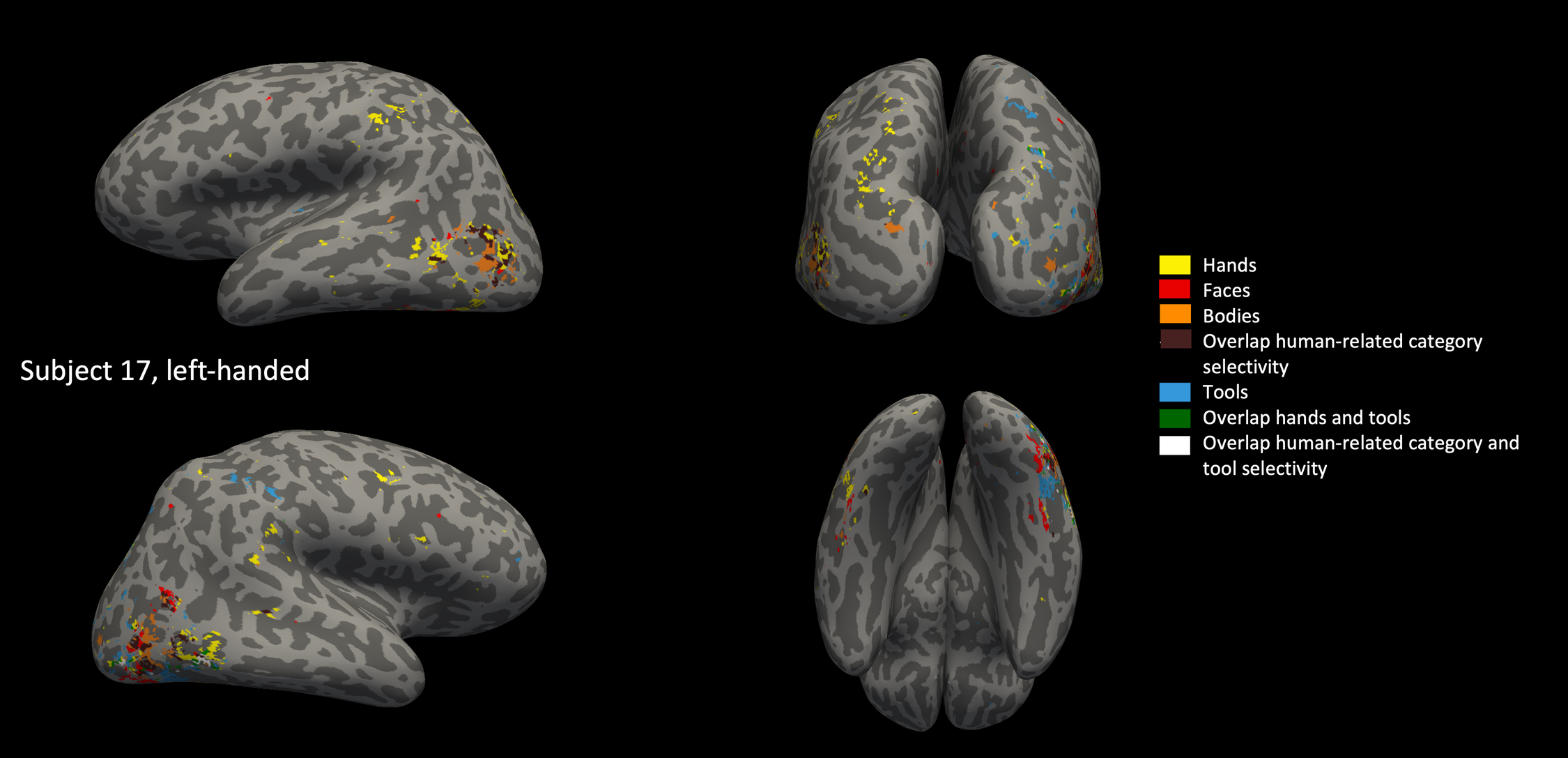

Supplement: S6 Fig — Hand, face, body and tool areas and their overlap, determined by a contrast of one versus all other categories except fixation, p < 0.05, FWE corrected (hands in yellow, faces in red, bodies in orange, overlap between some or all of these three animate categories in brown, tools in blue, overlap between hands and tools in green, and between animate categories and tools in white), shown upon annotated left lateral (top left), posterior (top right), right lateral (bottom left) and ventral (bottom right) brain surface of participant 17 (color legend also on the right of the figure). (TIF) [file pone.0308565.s007.tif]

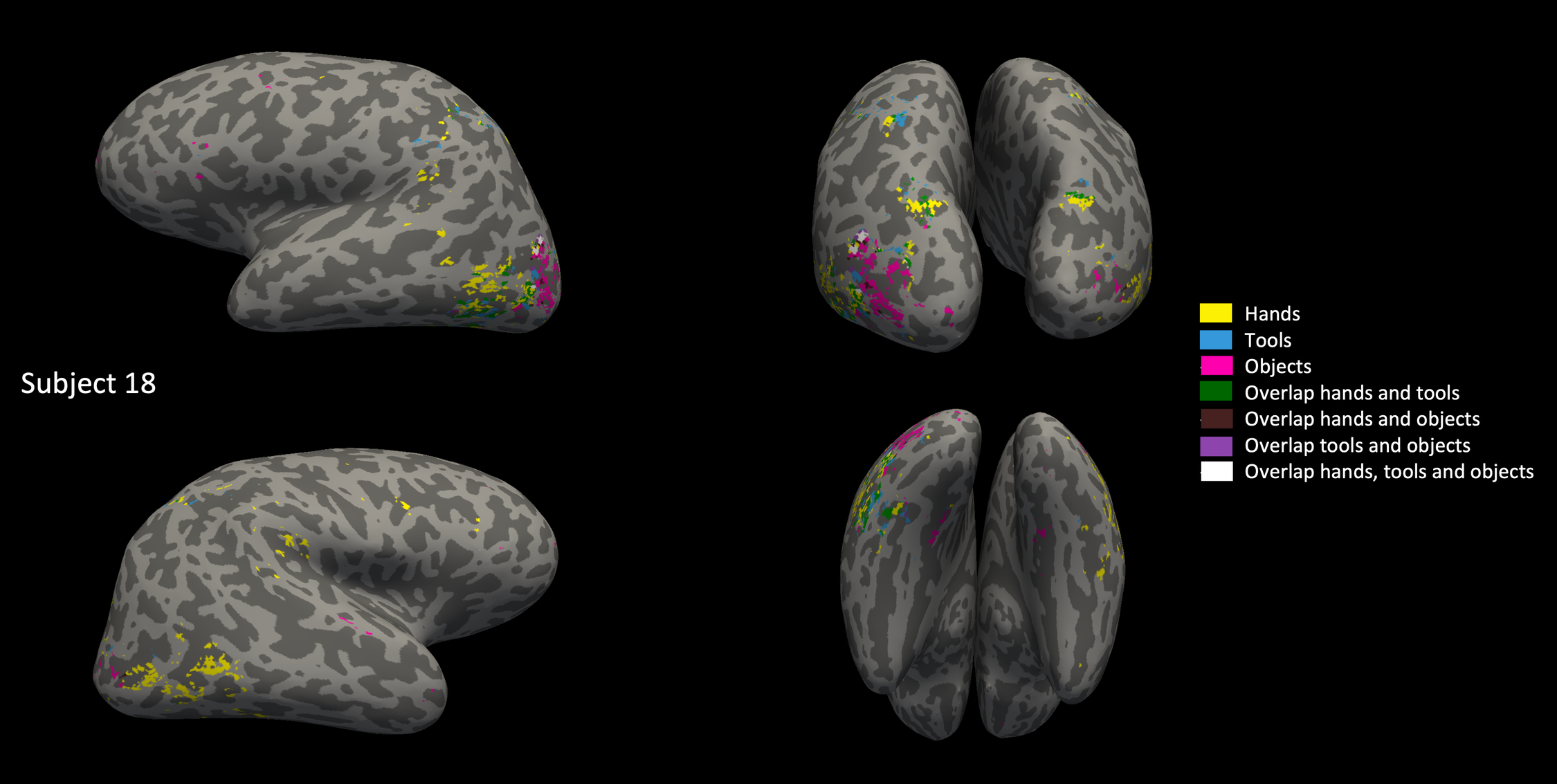

Supplement: S7 Fig — Hand, tool and object areas and their overlap, determined by a contrast of one versus all other categories except fixation, p < 0.05, FWE corrected (hands in yellow, tools in blue, objects (chairs) in pink, overlap between hands and tools in green, between hands and objects in brown, between tools and objects in purple and between hands, tools and objects in white), shown upon annotated left lateral (top left), posterior (top right), right lateral (bottom left) and ventral (bottom right) brain surface of participant 18 (color legend also on the right of the figure). (TIF) [file pone.0308565.s008.tif]

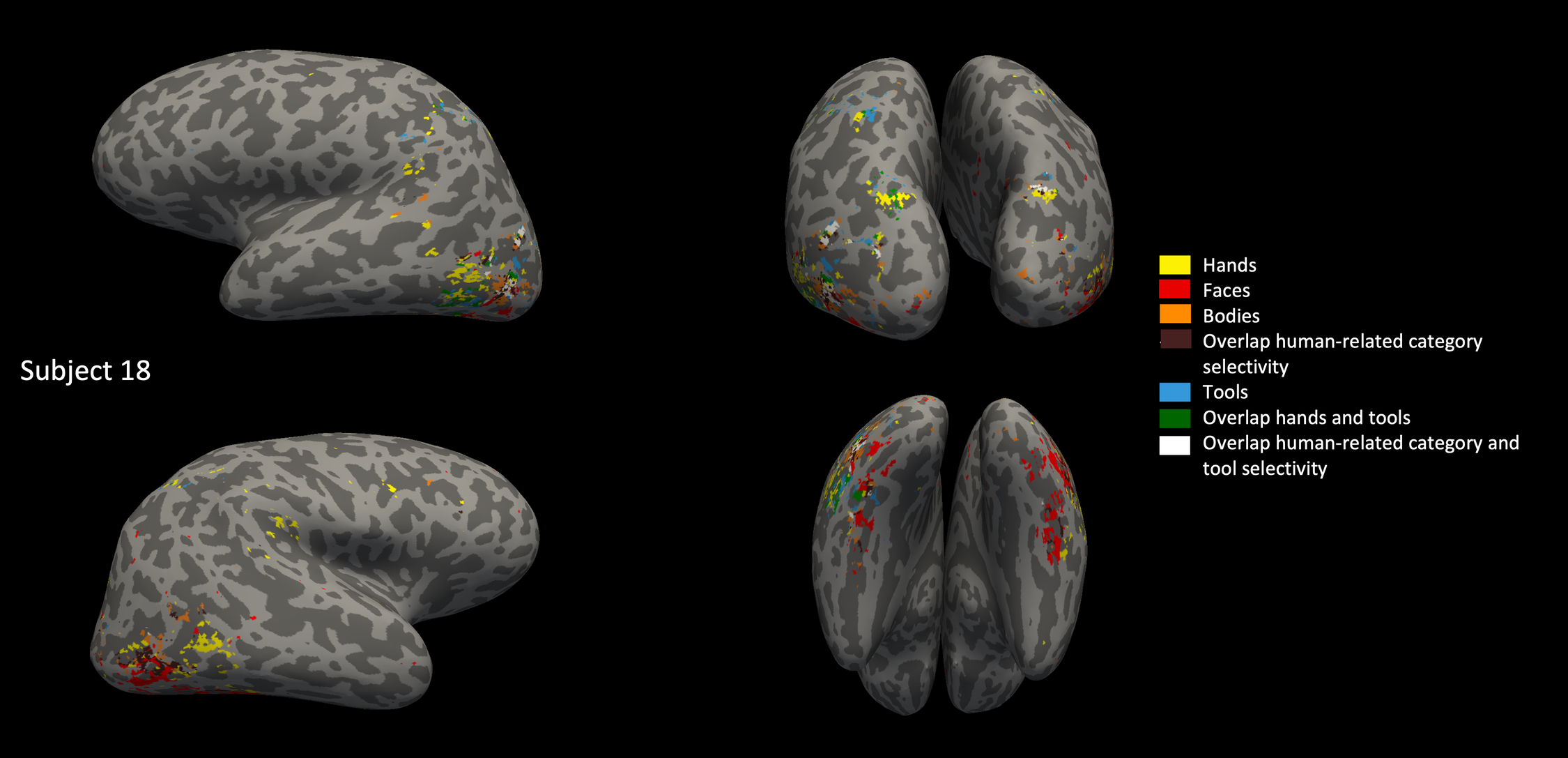

Supplement: S8 Fig — Hand, face, body and tool areas and their overlap, determined by a contrast of one versus all other categories except fixation, p < 0.05, FWE corrected (hands in yellow, faces in red, bodies in orange, overlap between some or all of these three animate categories in brown, tools in blue, overlap between hands and tools in green, and between animate categories and tools in white), shown upon annotated left lateral (top left), posterior (top right), right lateral (bottom left) and ventral (bottom right) brain surface of participant 18 (color legend also on the right of the figure). (TIF) [file pone.0308565.s009.tif]
